# Optimising weight-loss interventions in cancer patients—A systematic review and network meta-analysis

**DOI:** 10.1371/journal.pone.0245794

**Published:** 2021-02-04

**Authors:** Nathalie LeVasseur, Wei Cheng, Sasha Mazzarello, Mark Clemons, Lisa Vandermeer, Lee Jones, Anil Abraham Joy, Pauline Barbeau, Dianna Wolfe, Nadera Ahmadzai, Mona Hersi, Carol Stober, Risa Shorr, John Hilton, Brian Hutton

**Affiliations:** 1 Division of Medical Oncology, British Columbia Cancer Agency, Vancouver, Canada; 2 Clinical Epidemiology Program, Ottawa Hospital Research Institute, Ottawa, Canada; 3 Ottawa Hospital Research Institute, Ottawa, Canada; 4 Memorial Sloan Kettering Cancer Center, New York, New York, United States of America; 5 Weill Cornell Medical Center, New York, New York, United States of America; 6 Division of Medical Oncology, Department of Oncology, University of Alberta, Cross Cancer Institute, Edmonton, Canada; 7 Ottawa Hospital, Ottawa, Canada; 8 University of Ottawa School of Epidemiology and Public Health, Ottawa, Canada; University of Nebraska Medical Center, UNITED STATES

## Abstract

**Background:**

Excess weight has been associated with increased morbidity and a worse prognosis in adult patients with early-stage cancer. The optimal lifestyle interventions to optimize anthropometric measures amongst cancer patients and survivors remain inconsistent.

**Objective:**

To conduct a systematic review and network meta-analysis (NMA) of randomized controlled trials (RCTs) comparing the effects of exercise and dietary interventions alone or in combination on anthropometric measures of adult cancer patients and survivors.

**Methods:**

A systematic search of Medline, Embase and the Cochrane Trials Registry was performed. Outcomes of interest included changes in weight, body mass index (BMI), and waist circumference. Screening and data collection were performed by two reviewers. Bayesian NMAs were performed.

**Results:**

Overall, 98 RCTs were included; 75 were incorporated in NMAs (n = 12,199). Groups of intervention strategies included: 3 exercise interventions, 8 dietary interventions, 7 combination interventions of diet and exercise and standard care. Median intervention duration was 26 weeks. NMA suggested that diet alone (mean difference [MD] -2.25kg, 95% CrI -3.43 to -0.91kg) and combination strategies (MD -2.52kg, 95% CrI -3.54 to -1.62kg) were associated with more weight loss compared to standard care. All dietary interventions achieved a similar magnitude of weight loss (MD range from -2.03kg to -2.52kg). Both diet alone and combination strategies demonstrated greater BMI reductions versus standard care, and each of diet alone, exercise alone and combination strategies demonstrated greater reductions in waist circumference than standard care.

**Conclusion:**

Diet and exercise alone or in combination are effective lifestyle interventions to improve anthropometric measures in cancer patients and survivors. All reputable diets appear to be similarly effective to achieve weight loss.

## Background

Being either overweight (body mass index (BMI) between 25–29.9 kg/m^2^) or obese (BMI >30 kg/m^2^) contributes to increased cancer-related mortality [1–4]. In addition, excessive energy intake and suboptimal levels of physical activity may influence the course of the disease, treatment efficacy and toxicity, as well as overall health, well-being and overall survival [5–12]. The importance of addressing obesity in cancer patients was demonstrated by the 2014 American Society of Clinical Oncology (ASCO) position statement outlining their commitment to promoting research delineating the relationship between obesity and cancer, as well as educating oncology care providers about best practices for weight control [13]. Subsequently, a summit on Advancing Obesity Clinical Trials in Cancer Survivors was convened to provide recommendations and highlighted the need to further evaluate the impact of energy balance on cancer outcomes and define the degree of benefit in various cancer survivor subgroups [14].

Studies in cancer patients and survivors have demonstrated that lifestyle changes such as increased physical activity and improved dietary quality can lead to weight control and weight loss [15–18]. However, these interventions include a broad range of dietary interventions (e.g. low-fat, hypocaloric, and Mediterranean) and physical activity (e.g. aerobic and resistance training). Despite the rapidly increasing number of trials supporting the safety of exercise and dietary interventions in the adjuvant and post-treatment settings [19–29], the optimal method to mitigate weight gain during cancer treatment and to achieve weight loss after treatment, across various early-stage cancers, has yet to be identified [14, 15]. While systemic reviews looking at the role of diet and exercise on weight in specific cancer subgroups have been undertaken [17, 18, 30–33], no such reviews have directly compared the effect of various diet and exercise interventions to each other, alone or in combination, on anthropometric measures across various cancer types [34–36]. Additionally, guidelines regarding weight management strategies have not focused on cancer patients and survivors [13].

The purpose of this systematic review was to explore and synthesize the available evidence evaluating the optimization of anthropometric measures through various diet, exercise and combination interventions, during and after cancer treatment, in adult patients with early stage cancer. In turn, this will allow for the identification of optimal intervention recommendations to address obesity in cancer patients and survivors, as well as determining gaps in the evidence that should be addressed by future research.

## Methods

A protocol for the study was prepared a priori and followed throughout the review process; this protocol is provided in S1 Text. The PRISMA Extension Statement for Network Meta-Analysis was used to guide the reporting of this study [37].

### Study question and inclusion criteria

This systematic review was designed to identify and synthesize the available data addressing the following research question framed in the Population-Intervention-Comparator-Outcome-Study Design (PICOS) framework: “*Based on data from randomized controlled trials*, *what are the relative effects of competing weight loss strategies in terms of changes in anthropometric measures for patients with early stage cancer*?*”* The population of interest was adult cancer patients included in trials during and after treatment for early stage cancer of any type. Studies aiming to mitigate weight-loss or cachexia during cancer treatment were excluded. Studies including patients with advanced cancer were also excluded. The interventions of interest were lifestyle intervention strategies in the form of dietary interventions, exercise interventions and combinations thereof. All diet and exercise plans and comparisons versus standard of care were of interest. Interventions primarily leveraging counselling or behavioural therapy, and interventions comparing the method of intervention delivery were only included in the qualitative analysis. Outcomes of interest included change in body weight (in kilograms), body mass index (BMI), and waist circumference (WC). Only randomized studies were sought, with no restrictions on duration of intervention or patient follow-up. The longest available follow-up data while enrolled was used for data extraction.

### Literature search

An information specialist (RS) designed and performed an electronic literature search to identify relevant articles. Ovid Medline (1946-September 2019), EMBASE (1947-September 2019) and the Cochrane Central Register of Controlled Trials (1993–September 2019) were searched to identify relevant citations. The search consisted of key terms (e.g. *weight management*, *weight loss*, *energy intake*, *diet*, *exercise*), related text word searches and was limited to English language studies. The reference sections of included papers were reviewed to identify additional relevant citations. The full literature search strategy is provided in S2 Text and was peer-reviewed using the PRESS framework by a second information specialist [38].

### Study screening and study selection

Pairs of reviewers from the authorship team (NL, SM, MC, LV, CS, JH, AAJ) screened all citations independently. Stage 1 review consisted of screening titles and abstracts only. Stage 2 review consisted of screening the full texts of citations that were considered potentially relevant. After each stage reviewers (NL, SM, MC, LV, CS, JH, AAJ) resolved discrepancies through a third party if needed. The process of study selection is presented in a flow diagram [39].

### Data collection and risk of bias assessment

Data collection from the included studies was performed by two reviewers (NL, SM) using a standardized data extraction template implemented in Microsoft Excel (version 10, Microsoft Corporation, Seattle, Washington, USA). A pilot test of the data collection form was performed on the first 5 studies and refined accordingly. Data items collected included the following: study design, patient eligibility criteria, patient demographics (e.g. type of malignancy, performance status, age distribution, menopausal status, and baseline measures of weight, BMI and waist circumference), intervention details (diet, exercise or combination regimen, along with corresponding details of each intervention), and outcome data (final values and/or changes in body weight, BMI, waist circumference). After data collection, the reviewers resolved any discrepancies and consulted a third party when needed.

Full text articles were independently assessed for risk of bias by 2 reviewers. The Cochrane Collaboration’s tool for assessing risk of bias in randomized trials was used [40]. The tool assessed potential areas of bias including selection bias, performance/detection bias, attrition bias and reporting bias. Discrepancies in the initial independent assessments were resolved by discussion. A narrative summary of findings from these assessments is provided in the main text, while a tabular summary of all assessments is provided in the review supplement.

### Structuring the evidence networks for meta-analysis

There was interest to compare the effects of specific interventions to each other (e.g. to compare different types of diets head-to-head, or against different exercise or combination strategies) and to compare groups of clinical relevance (i.e. standard care therapy versus exercise interventions versus dietary interventions versus combined interventions). An NMA model allowing for comparisons at both levels (intervention level and group level) in the same analysis was implemented [41]. For the more granular level of comparisons, in addition to standard care, there were 18 interventions, including those of *dietary interventions* (including low calorie diet, low carbohydrate diet, low fat diet, Mediterranean diet, NCI diet, phyto-rich/plant-based diet, low fat + low calorie diet, and low fat + phyto-rich diet), *exercise interventions* (aerobic exercise, resistance exercise, combined program of aerobic and resistance exercise), and *combined diet and exercise*; the number of interventions compared per analysis varied according to the number of studies with available outcome data. Group level comparisons involved estimated measures of effect between standard care, diet interventions, exercise interventions and combined diet and exercise interventions. An expert in exercise science and nutrition (LJ) established the optimal categorizations of therapies into the dietary, exercise and combination groups and at the individual intervention level. A practical approach was utilized to focus on the nature of the intervention without details about the specific restrictions or parameters of the intervention (e.g., exercise programs were grouped in terms of activity such as aerobic exercise or resistance training, but variations in frequency and duration were not modeled).

### Methods for evidence synthesis

NMA is an extension of traditional pairwise meta-analysis which enables the comparison of multiple interventions in a single analysis, and which allows for incorporation of both direct and indirect evidence of relevance [34, 35]. NMAs of the changes from baseline in body weight, body mass index and waist circumference were performed. The nature of reporting these endpoints varied across included studies, with some reporting changes from baseline while others reported mean values of each endpoint at baseline and follow-up, with standard deviations for each. For the latter, we calculated the mean changes from baseline and imputed the standard errors of the mean changes (for details, see S3 Text). We fit random effects (RE), three-level hierarchical models with a Normal likelihood and identity link [41] based on the mean changes from baseline and corresponding standard errors, with clustering of the interventions into 4 groups (standard care, diet interventions, exercise interventions and combined interventions); the main text focuses upon group level comparisons, while the intervention level comparisons are reported in detail in the report appendices. All mean differences (MD) of interventions versus standard care were reported along with corresponding 95% credible intervals. Forest plots are presented to summarize findings versus the standard care group, while all possible pairwise comparisons between interventions are summarized using league tables provided in this review’s online supplement. Details regarding our approach to model selection and fit assessment are also provided in the review supplement. The assumption of consistency between direct and indirect evidence was assessed by plotting the posterior mean deviance contributions from the consistency model against those from the unrelated means model to see if they aligned. All NMAs were performed using OpenBUGS software version 3.2.3 [42] and the R package R2OpenBUGS [43]. Model convergence was assessed using established methods including Gelman-Rubin diagnostics and the Potential Scale Reduction Factor [42, 44]. Findings reported within the main text of the review focus upon results from NMAs, while author conclusions of the remaining studies that did not appropriately fit into the NMAs (due to the types of comparisons made or lack of sufficient data) are summarized in the appendices. The Comparison-adjusted funnel plots were applied to assess for small-study effects as signals of publication bias. Following presentation of detailed supporting information for all results from the review (S4–S7 Text), the OpenBUGS code for data analyses is provided in S8 Text, while raw data used for NMAs for the outcome measures of weight change, BMI change and waist circumference change are provided in S1, S2 and S3 Data, respectively. A completed PRISMA NMA Checklist is provided in S9 Text.

## Results

### Quantity of evidence identified

**Fig 1:**
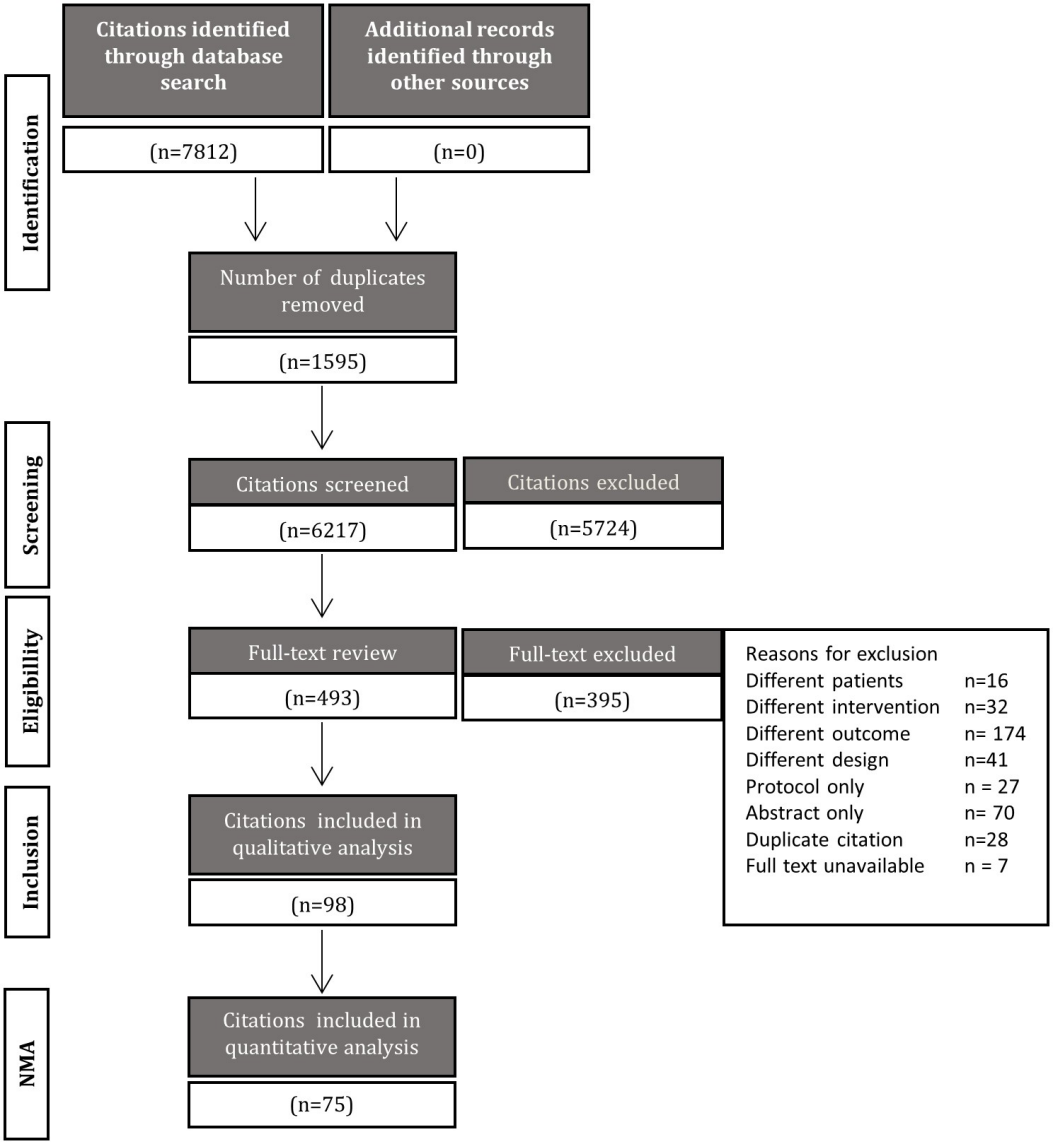
Process of study selection. A flow diagram is shown which depicts the process of study selection.

The initial search identified 7,812 articles. Duplicates were removed (n = 1,595), leaving 6,217 unique citations for review. Stage 1 screening of titles and abstracts identified 493 potentially relevant citations, which were subsequently reviewed in full text. Of these citations, 98 met the a priori inclusion criteria [20, 24, 45–142], representing 75 studies that were included for analysis [20, 24, 45–51, 53–98, 100–108, 110–112, 114–121] (Table 1). Reasons for study exclusion are listed in the flow diagram presented in Fig 1. A list of studies identified as meeting eligibility criteria but not included in NMAs is provided in S4 Text, with supporting rationale for their exclusion from syntheses of the data. For reasons related to network structure, the research was not planned to consider differences in method of delivery (n = 4) [123, 130, 132, 136] or intensity (n = 2) [125, 127]. Behavioral and counselling therapies were included in the qualitative analyses, but not considered of interest for the NMAs (n = 6) [122, 124, 131, 133, 139, 141]. Interventions used in only 1 study were excluded from the network (n = 7) [52, 113, 128, 134, 135, 137, 138]. Studies missing data also precluded NMA and were excluded (n = 3) [109, 129, 142].

**Table 1:** Overview of study characteristics included in NMAs (n = 75).

Characteristic	Summary Measure
**Year of publication**	
2010>	59 (78.7%)
2001–2009	16 (21.3%)
1991–2000	0 (0%)
<1990	0 (0%)
**Study sample size**	
<50 patients	33 (44.0%)
51–100 patients	27 (36.0%)
101–500 patients	13 (17.3%)
501–1000 patients	0 (0%)
>1000 patients	2 (2.7%)
**Time of Study Intervention**	
Pre-operative	3 (4.0%)
During chemotherapy	9 (12.0%)
During adjuvant hormone therapy/androgen deprivation	9 (12.0%)
After treatment	54 (72.0%)
**Type of cancer patients enrolled**	
Breast	48 (64.0%)
Prostate	13 (17.3%)
Colorectal	3 (4.0%)
Mixed sites	8 (10.6%)
Other sites	3 (4.0%)
**Median of average patient ages (range)**	57 (42–73)
**Median of average patient body weights (range kg)**	80 (49–98)
**Median of average patient BMI (range kg/m**^**2**^**)**	29 (23–35)
**Duration of study intervention**	
<3 months	14 (18.7%)
3–6 months	44 (58.7%)
7–12 months	11 (14.7%)
>12 months	5 (6.7%)
Not reported	1 (1.3%)
**# studies involving a treatment group of**:	
Standard care	72 (96.0%)
Dietary Intervention	16 (21.3%)
Exercise Therapy	45 (60.0%)
Combination Intervention	26 (34.7%)

### Study characteristics, patient characteristics and risk of bias

Amongst data from a total of 14,378 patients in the included studies, 12,199 were included in NMAs. Study characteristics including year of publication, study size, duration, type of malignancy, median age, weight and BMI are summarized in Table 1, while a detailed intervention-level description is provided in S5 Text. Individual study sizes ranged from 10 [64] to 3,088 [76]. The studies enrolled patients with a range of tumour types including breast cancer (48 studies, 9,513 patients) [20, 24, 56, 58, 59, 61, 62, 64–71, 73–80, 82, 88, 89, 91, 93–97, 102, 103, 105, 106, 108, 110, 112, 114, 115, 117, 119, 143], prostate cancer (9 studies, 521 patients) [83–85, 90, 92, 101, 107, 111, 118], colorectal cancer (3 studies, 301 patients) [104, 116, 120], mixed tumour sites (primarily breast, prostate and colorectal) (8 studies, 1,812 patients) [60, 63, 72, 81, 100, 121] and other sites including endometrial and lung cancer (3 studies, 181 patients) [86, 87, 98]. Risk of bias assessment of the included studies showed that few of the studies concealed treatment allocation and many had an unknown risk for blinding to outcome (detailed assessments are provided in S5 Text).

### Intervention characteristics and outcomes reported

**Fig 2:**
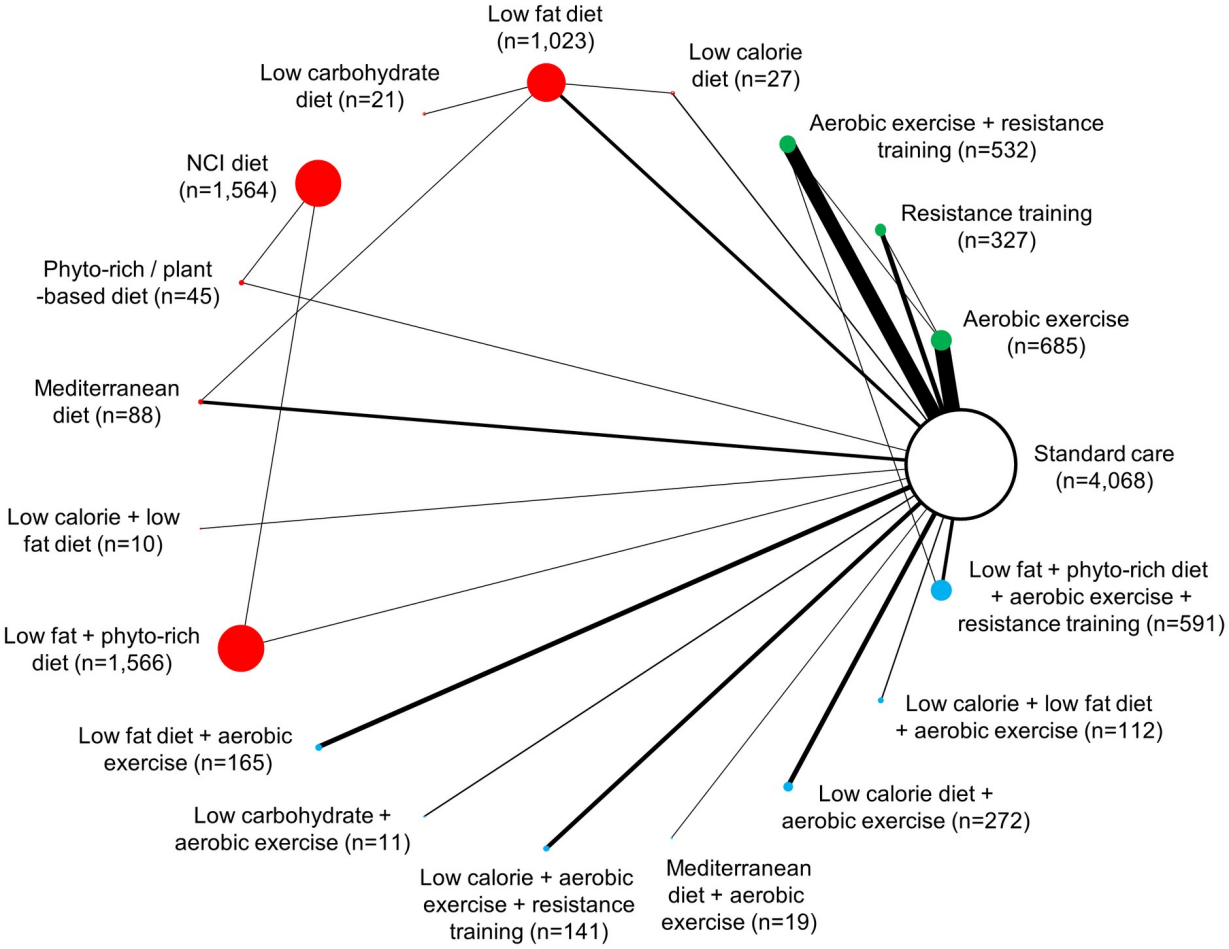
Network diagram for change in body weight (kg): 18 interventions and standard care, 11,267 patients. The evidence network of the available studies and interventions for change in body weight is shown. Joining lines denote intervention comparisons where one or more trials were available. Nodes are proportionally sized to reflect the numbers of patients studied with each intervention. Edge width reflects the number of RCTs for each comparison. Nodes coloured green represent interventions considered to belong to the exercise group, while red nodes reflect the dietary group and the blue node denotes the diet/exercise combination group.

**Fig 3:**
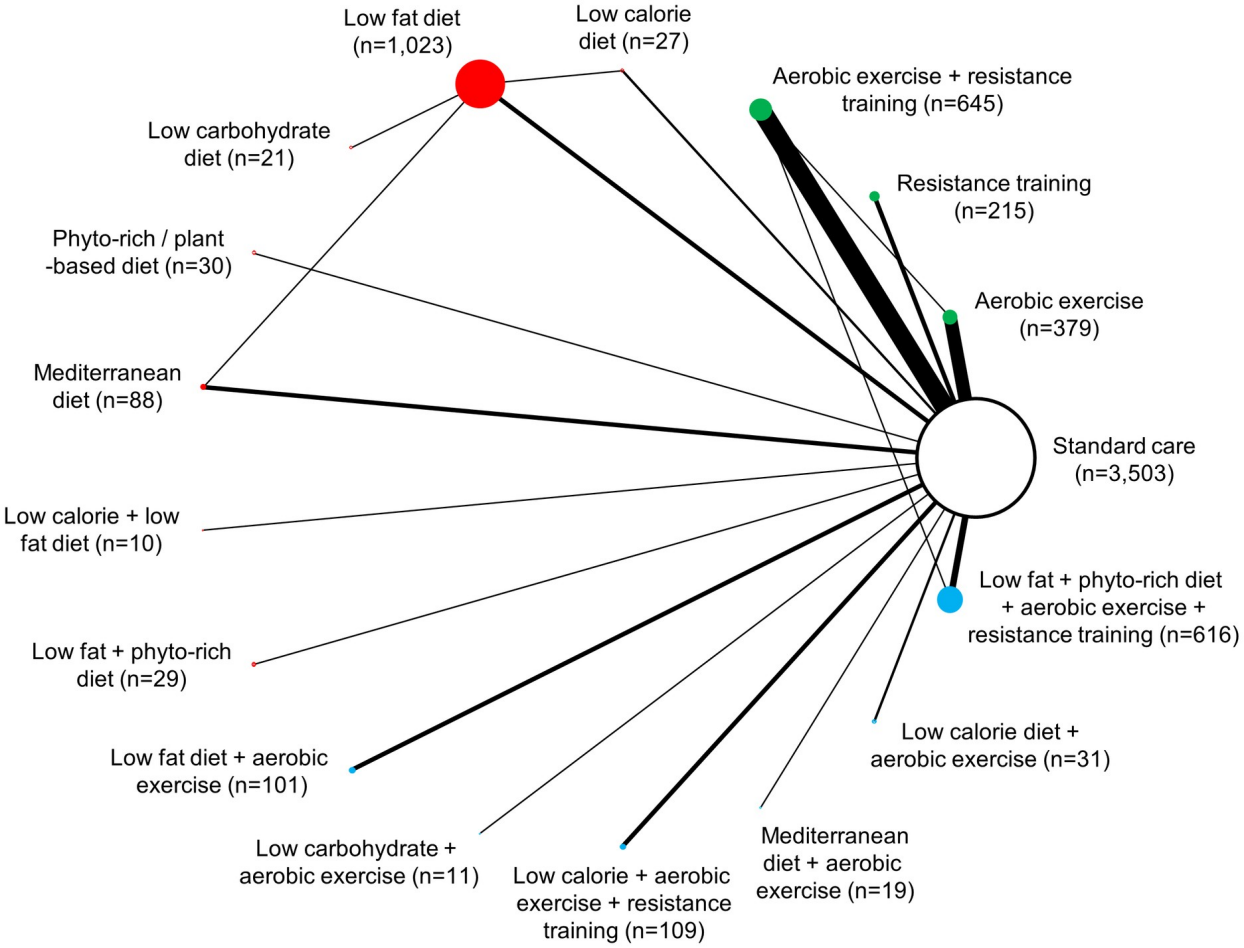
Network diagram for change in BMI: 16 interventions and standard care, 6,857 patients. The evidence network of the available studies and interventions for change in BMI is shown. Joining lines denote intervention comparisons where one or more trials were available. Nodes are proportionally sized to reflect the numbers of patients studied with each intervention. Edge width reflects the number of RCTs for each comparison. Nodes coloured green represent interventions considered to belong to the exercise group, while red nodes reflect the dietary group and the blue node denotes the diet/exercise combination group.

**Fig 4:**
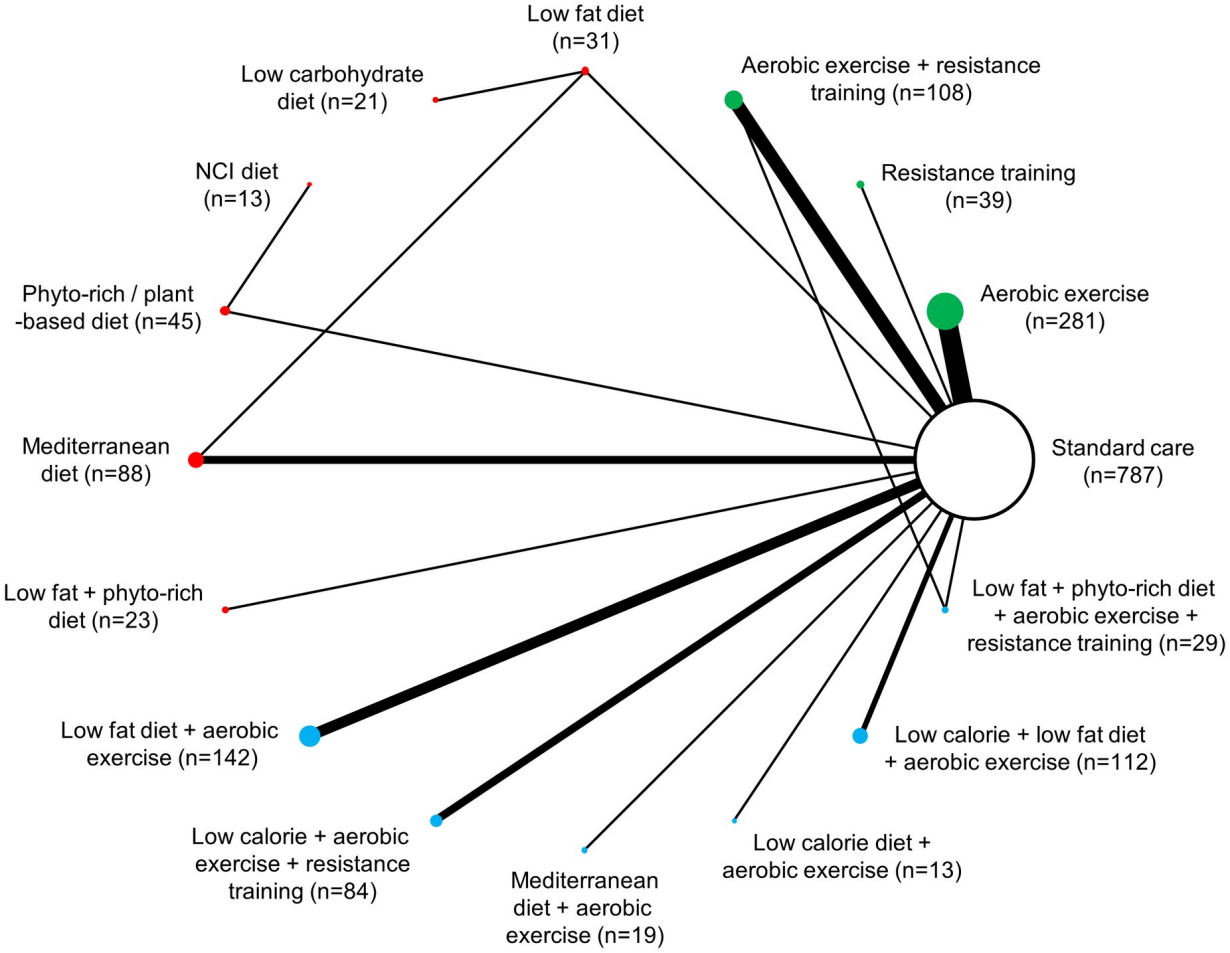
Network diagram for change in waist circumference: 15 interventions and standard care, 1,835 patients. The evidence network of the available studies and interventions for change in waist circumference is shown. Joining lines denote intervention comparisons where one or more trials were available. Nodes are proportionally sized to reflect the numbers of patients studied with each intervention. Edge width reflects the number of RCTs for each comparison. Nodes coloured green represent interventions considered to belong to the exercise group, while red nodes reflect the dietary group and the blue node denotes the diet/exercise combination group.

Overall, studies included in NMAs evaluated dietary interventions (n = 11) [20, 56, 65, 68, 69, 76, 77, 80, 84, 106, 115], exercise interventions (n = 36) [25, 57, 60, 64, 66, 67, 70, 71, 73–75, 78, 81–83, 89, 92, 93, 95, 97, 98, 100, 101, 103, 104, 107, 110–112, 114, 116, 118, 120, 129, 140, 144] or a combination of both dietary and exercise interventions (n = 21) [58, 59, 61–63, 72, 74, 85–88, 90, 91, 94, 96, 102, 105, 108, 117, 119, 121]. Change in body weight was reported by 65 studies (n = 11,267), while changes in BMI and waist circumference were reported by totals of 47 studies (n = 6,875) and 31 studies (n = 1,835), respectively (S5 Text). Figs 2–4 present network diagrams displaying the patterns of comparisons and numbers of patients per intervention for each endpoint assessed using NMA. The majority of comparisons in the included studies used standard care as the control group; standard care across studies generally consisted of information handouts related to food intake, while small numbers of studies involved waitlist controls or were lacking description. The number of interventions per NMA varied from a maximum of 18 for the weight loss endpoint to 15 for waist circumference. The total numbers of studies (minimum 31 for waist circumference to maximum 65 for body weight) and patients (from minimum 1,835 for waist circumference to maximum 11,267 for body weight) also varied notably across analyses based on availability of data.

### NMA model fit evaluation for analyses of body weight, BMI and waist circumference

For all three endpoints, comparison of posterior residual deviance values with the numbers of unconstrained data points indicated adequate model fit of random effects models, which were found to be preferred to fixed effects analyses based on comparison of DIC values (see review supplement, S6 Text for details). Inspection of DIC values and posterior mean deviance contributions did not identify evidence that the consistency assumption for NMA was violated for any of the RE NMAs, and thus results from RE consistency models are the focus of the report. There was no evidence of publication bias based on comparison adjusted funnel plots (see S7 Text).

### Findings from NMA: Weight change

**Fig 5:**
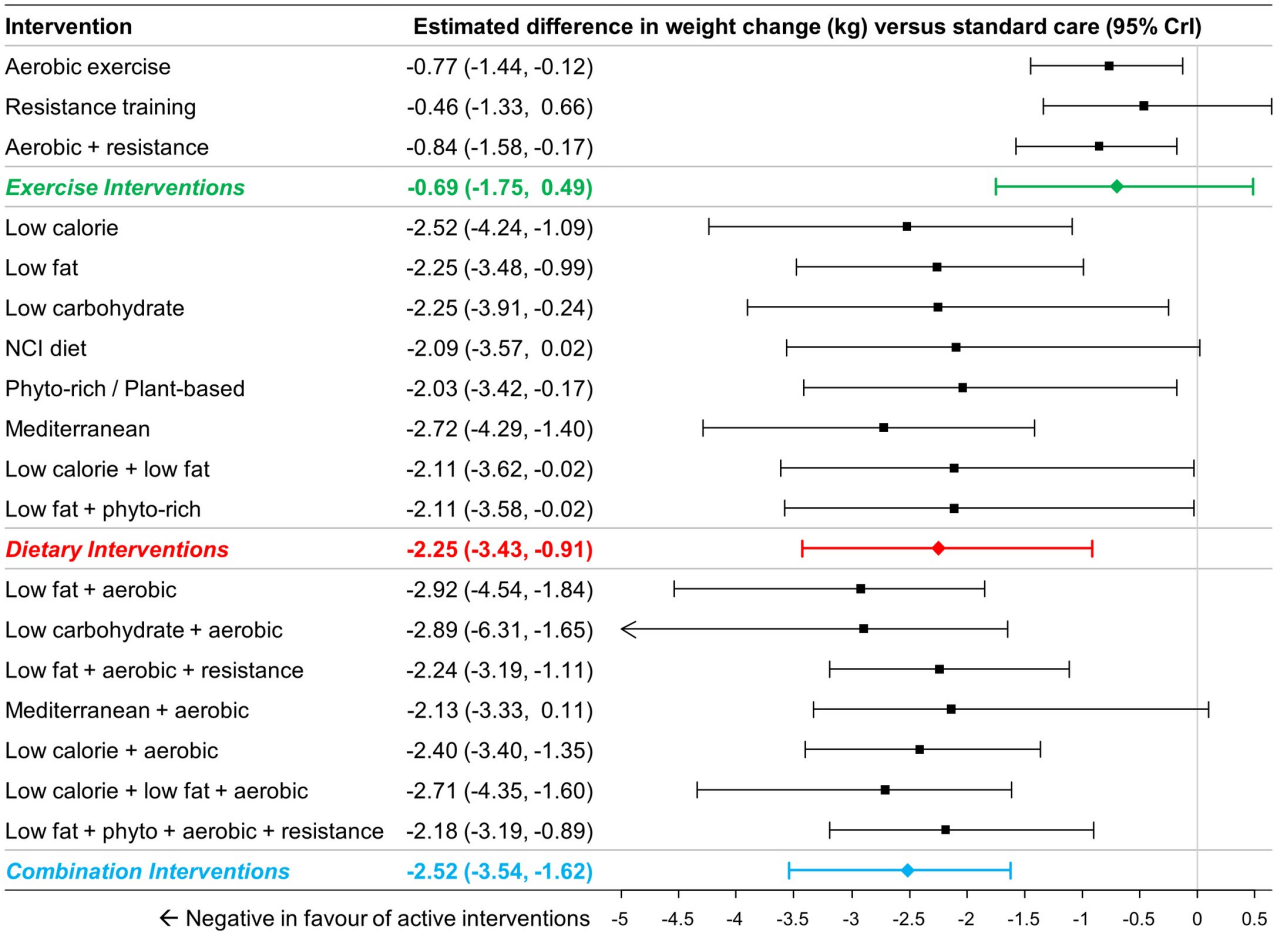
Estimated differences in weight change (kg) compared to standard care from NMA (18 interventions and standard care, 65 studies, 11,267 patients). The estimated differences (2.5% and 97.5% quantiles) of interventions versus standard care from random effects consistency model are displayed. Colored summary estimates represent estimated treatment effects of the groups of interventions versus standard care.

A total of 80 studies (13,069 patients) reporting weight change were identified [20, 24, 45, 47–50, 52–54, 56, 58–63, 65, 66, 68–72, 74–89, 91–96, 99, 101–112, 115–121, 123, 125, 127, 128, 130, 131, 134–136, 138, 140–143]. A total of 65 studies (11,267 patients) comprising 18 interventions (and standard care) were included in the NMAs of changes in weight from baseline measured in kilograms [20, 24, 45, 47–51, 53, 54, 56, 58–63, 65, 66, 68–72, 74–89, 91–96, 101–108, 110–112, 115–121, 145]. Fig 5 summarizes the estimates from NMA for group comparisons versus standard care, while the online supplement provides numerical details of pairwise comparisons.

For comparisons at the group level, pairwise comparisons versus standard care found dietary interventions (MD -2.25kg, 95% CrI –3.43 to -0.91kg) and combination interventions (MD -2.52kg, 95% CrI -3.54 to -1.62kg) to be associated with statistically significantly greater weight reductions, while exercise interventions (MD -0.69kg, 95% CrI -1.75 to +0.49kg) were not (Fig 5). Comparisons between the different groups found both dietary interventions (MD -1.56kg, 95% CrI -3.12 to +0.17kg) and combination interventions (MD -1.82kg, 95% CrI -3.43 to -0.50kg) to be associated with greater weight loss than exercise interventions. Changes with dietary and combination interventions were similar (MD -0.26kg, 95% CrI -2.04kg to +1.19kg).

For comparisons at the intervention level, all 8 dietary interventions were associated with greater reductions in body weight when compared to standard care with all differences being of similar magnitude (range -2.03 to -2.52kg; Fig 5). Most combined interventions demonstrated a comparable difference in weight change versus standard care (range -2.13 to -2.92kg).

### Findings from NMA: BMI change

**Fig 6:**
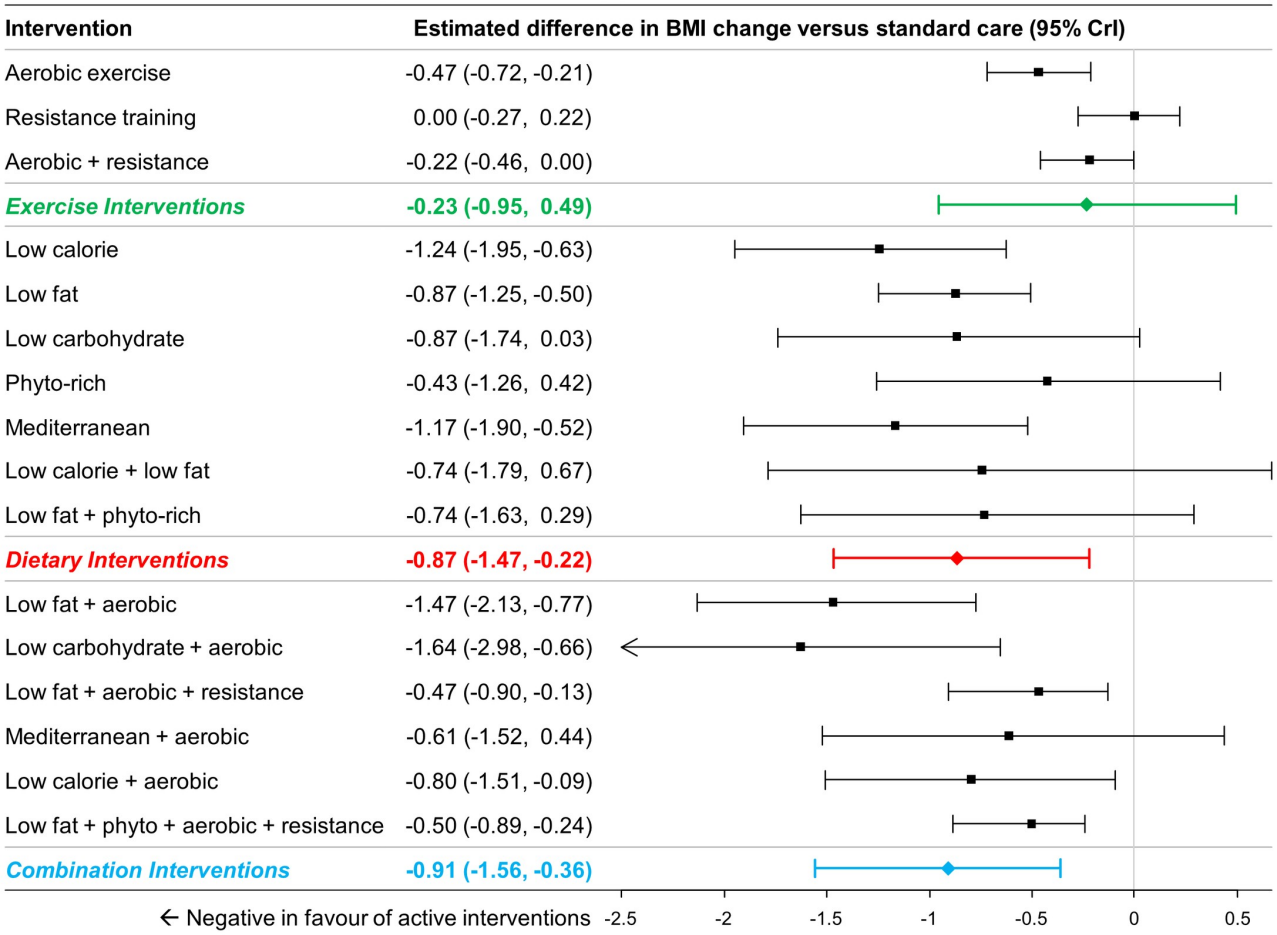
Estimated differences in BMI change compared to standard care from NMA (16 interventions and standard care, 47 studies, 6,824 patients). The estimated differences (2.5% and 97.5% quantiles) of interventions versus standard care from random effects consistency model are displayed. Colored summary estimates represent estimated treatment effects of the groups of interventions versus standard care.

A total of 58 studies (7967 patients) were identified [20, 24, 45–48, 50, 53–56, 58, 60, 62–65, 69–74, 77, 79, 80, 84, 85, 88, 90, 92–102, 106, 108, 110, 111, 113–116, 122, 131–134, 136–140] and a total of 39 studies comprising 15 interventions (and standard care) and 6,265 patients were included in for analysis [20, 24, 45–48, 50, 51, 53–56, 58, 60, 62–65, 69–74, 77, 79, 80, 84, 85, 88, 90, 92–98, 100–102, 106, 108, 110, 111, 114–116]. Two interventions that were included in the previous analysis of weight change had no available data for change in BMI (NCI diet, and low calorie / low fat diet combined with aerobic exercise). Comparisons at the group level found that dietary interventions (MD -0.87 kg/m^2^, 95% CrI -1.47 kg/m^2^ to -0.22 kg/m^2^) and the combination of diet with exercise interventions (MD -0.91 kg/m^2^, 95% CI -1.56 kg/m^2^ to -0.36 kg/m^2^) were associated with a greater BMI reduction compared to standard care, while exercise interventions were not (MD -0.23 kg/m^2^, 95% CrI -0.95 kg/m^2^ to 0.49 kg/m^2^) (Fig 6). Comparisons between dietary, exercise and combination of diet with exercise interventions revealed no significant differences (see S7 Text for numeric details).

Comparisons at the intervention level of the analysis found that aerobic exercise and aerobic exercise combined with resistance training showed significant differences in BMI reduction relative to standard care, but resistance training alone did not. All dietary interventions in the network were associated with BMI reductions (ranged -0.56 kg/m^2^ to -1.17 kg/m^2^), and differences were statistically significant for low calorie diet, low fat diet, low carbohydrate diet, and Mediterranean diet (Fig 6). Most combination interventions were also associated with statistically significant BMI reduction (except for aerobic exercise combined with Mediterranean diet).

### Findings from NMA, waist circumference change

A total of 39 studies included (2,616 patients) reported data on changes in waist circumference [56, 58, 60, 61, 65, 67, 68, 70, 71, 74, 79, 85, 86, 88, 89, 91, 94, 99, 102, 106, 108, 109, 112, 115, 116, 119, 120, 123, 127, 130, 131, 134, 136, 139, 140]. A total of 31 studies involving 15 interventions (and standard care) and 1,835 patients were included for analysis [46, 49–51, 53, 56, 58, 60, 61, 65, 67–71, 74, 79, 85, 86, 88, 89, 91, 94, 102, 106, 108, 112, 115, 116, 119, 120]; in comparison to the set interventions that were evaluated earlier for their effects on weight loss, no data were available for low calorie diet, NCI diet or low carbohydrate/low fat diet.

**Fig 7:**
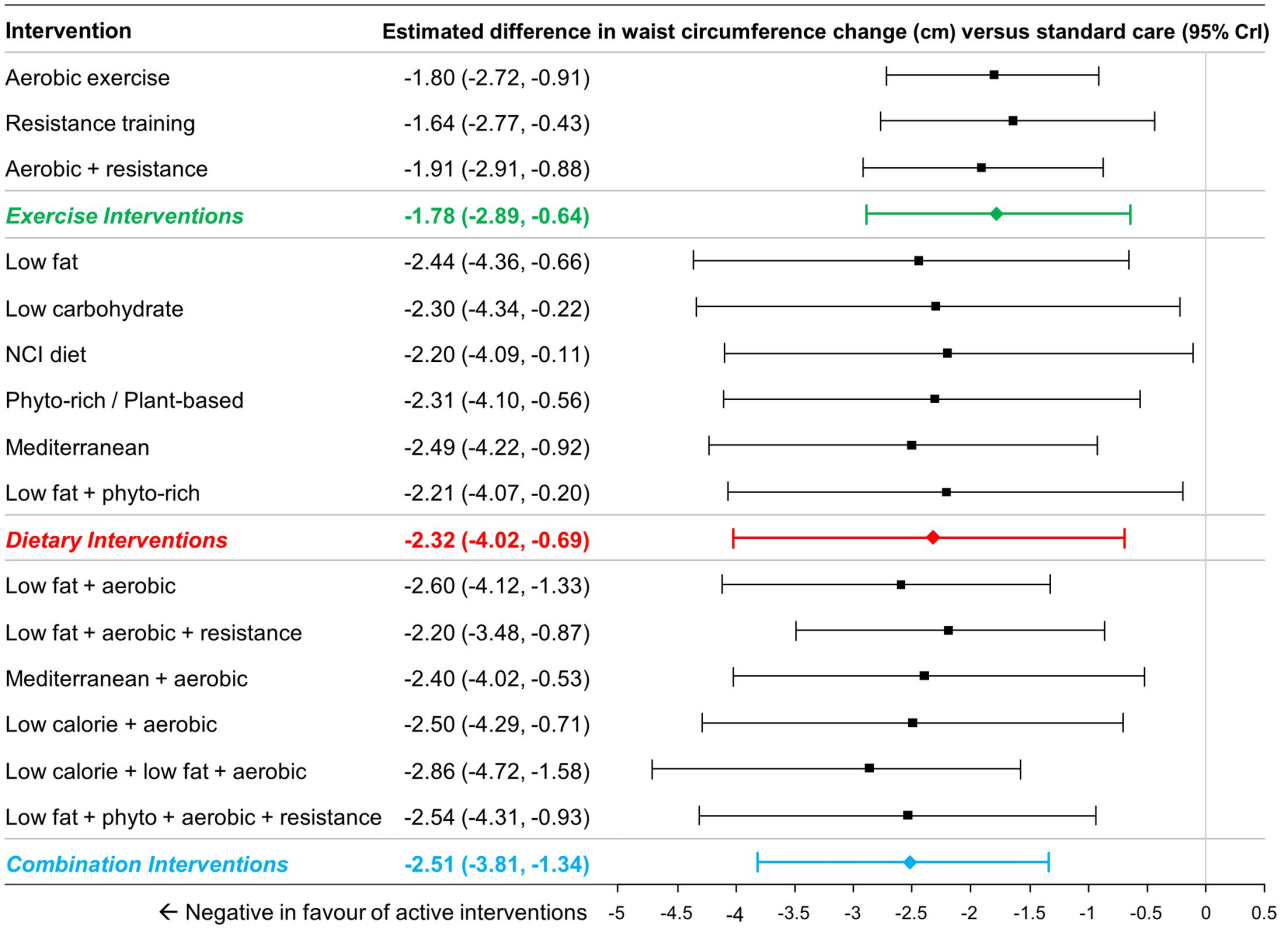
Estimated differences in waist circumference change (cm) compared to standard care from NMA (15 interventions and standard care, 32 studies, 1,875 patients). The estimated differences (2.5% and 97.5% quantiles) of interventions versus standard care from random effects consistency model are displayed. Coloured summary estimates represent estimated treatment effects of the groups of interventions versus standard care.

At the group level of the analysis, the groups of exercise interventions (MD -1.78cm, 95% CrI -2.89cm to -0.64cm), dietary interventions (MD -2.32cm, 95% CrI -4.02cm to -0.69cm) and combination interventions (MD -2.51cm, 95% CrI -3.81cm to -1.34cm) were all associated with greater reductions in waist circumference compared to standard care (Fig 7). Comparisons between the different active groups suggested that there was no evidence of important differences between combination interventions and exercise interventions (MD -0.72cm, 95% CrI -2.45 to 0.82cm), between dietary interventions and exercise interventions (MD -0.53cm, 95% CrI -2.53cm to 1.44cm) or between combination interventions and dietary interventions (MD -0.19cm, 95% CrI -2.30cm to 1.85cm).

At the intervention level of the analysis, all interventions demonstrated larger reductions than standard care (Fig 7; range of differences from -1.54cm to -2.86cm).

**Fig 8:**
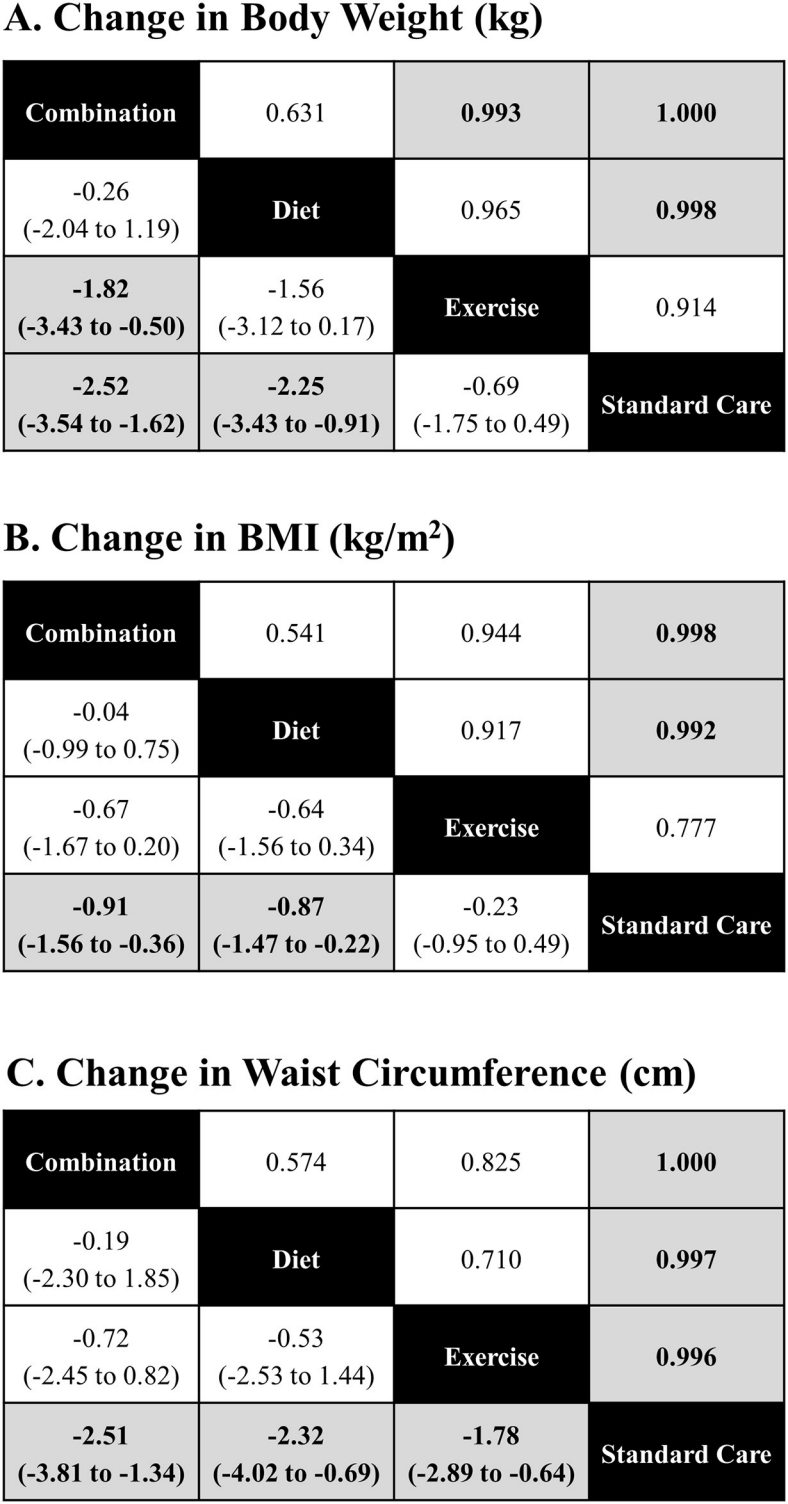
League tables, comparisons between group. League tables of estimated posterior median pairwise differences in weight change (kg) with credible intervals / 2.5% and 97.5% quantiles (lower triangle), and the pairwise probabilities that a treatment is better than another (upper triangle) are presented. A complete summary of estimates for efficacy from the RE consistency model assuming vague priors is displayed. Statistically significant differences between intervention categories are shown in bold, underlined font. The lower/right-most comparison for each comparison is the reference treatment.

Fig 8 (panels A, B and C) present league tables that summarize comparisons between intervention classes for each endpoint.

## Discussion

Our analyses reveal that dietary and combination regimens of diet and exercise during and after cancer treatment achieved greater weight, BMI and waist circumference reduction when compared to standard care in overweight and obese patients with early stage cancer. Moreover, changes in weight between eight different dietary interventions were of similar magnitude, highlighting the fact that dietary interventions with different macronutrient profiles achieve similar results. In addition, weight and BMI losses achieved with combination interventions were similar to dietary interventions alone, underlining the fact that dietary change has a significant effect on anthropometric measures. With regard to waist circumference, at the group level of analysis, exercise, dietary and combination interventions were all associated with significant reductions compared to standard care. However, the magnitude of change in waist circumference was most meaningful with combination interventions of diet and exercise.

There is increasing evidence of the negative impact of obesity in patients with breast [3, 7, 16, 146–150], ovarian [151], pancreatic [152], endometrial [153], prostate [154, 155] and colon cancer [156, 157]. It is clear that the obesity epidemic needs to be addressed with effective management strategies [13].

Oncology health care providers have not traditionally taken an active role in weight control management for their patients and resources directed at cancer patients and survivors seeking weight control and weight loss are limited [13]. This study highlights the fact that optimization of anthropometric measures in patients with early stage cancer is best achieved by dietary and combination interventions, but more importantly, that the improvement of anthropometric measures was similar, regardless of the specific lifestyle intervention used.

To our knowledge, this is the first systematic review comprising data from dietary, exercise and combination regimens together and comparing them at the group level and at the intervention level. An advantage of performing an NMA in this setting is that it is an extension of traditional pairwise meta-analysis, thus enabling the comparison of multiple interventions in a single analysis. It also permits the incorporation of both direct and indirect evidence of relevance [34, 35]. To proceed with analyses, pragmatic pooling of data into groups was performed in order to translate the findings of this study to clinical practice. Similar to previous meta-analyses on this topic, “standard care” arms were grouped together in order to facilitate the comparison.

While the results of our analyses have consistently identified dietary and combination regimens as having the greatest effect on weight, BMI and waist circumference, it is important to note that many of the studies included in this meta-analysis were not specifically designed to improve anthropometric measures, particularly for the studies comprising exercise interventions. It is therefore unclear if clinically meaningful results would be achieved if exercise studies were designed to improve anthropometric measures with exercise only. Further, gains in muscle mass are associated with an increase in lean body mass and decreased body fat, which may result in cumulative weight gain [158]. Body composition may therefore be a more meaningful endpoint to identify the benefits of lifestyle interventions. This endpoint was not included as part of this analysis as it is scarcely included in pragmatic lifestyle intervention trials and is of little use in the clinical oncology practice and survivorship care. Furthermore, ideal weight loss targets of clinical significance in the cancer population remain unknown. Many targets suggested have been derived from the Diabetes Prevention Programs [159] which recommend a 10% reduction in body weight, although this target is seldom achieved in lifestyle studies involving cancer patients. Additionally, although the evidence linking obesity to poor outcomes in observational studies is evident, the impact of changes in anthropometric measures on long-term outcomes remains uncertain [160]. Therefore, clinical trials attempting to identify achievable and sustainable targets for change in anthropometric measures which translate into improved outcomes are needed.

Amongst the limitations of this study, it should be noted that a practical approach to treatment classification was utilized which did not take into consideration granular details of interventions such as caloric or fat restrictions or intensity and frequency of exercise. This strategy was taken given that, while there were often minor differences in such details across studies, these differences were generally small. The interventions were not sufficiently well-connected in the evidence networks of weight, BMI and waist circumference. Therefore, our reporting of the results was focused on the group level rather the intervention level. It should also be noted that different intervention designs of efficacy, effectiveness or direct comparisons of the two may not necessarily translate into comparable clinically meaningful benefits. Variability existed in the definition of standard of care across studies and definitions of the standard of care arm was often limited or unclear. The median BMI of patients included in the studies was 29, with a range between 23 to 35. While this indicates that patients with a normal BMI were included in the studies utilized for meta-analyses, the majority of studies enrolled overweight or obese patients, limiting generalizability to patients with a BMI in the normal range. Finally, patients with early stage cancer of all types were included in this analysis to allow generalizability, but it should be noted that most patients enrolled in the presented studies had early stage breast, prostate and colorectal cancer. This study does not recognize the needs of specific patient populations that were not represented in the included studies, for whom losses could be detrimental, and should be interpreted with caution.

## Conclusions

This analysis reveals that dietary and combination interventions of diet and exercise targeted to overweight and obese patients with early-stage cancer significantly improved anthropometric measures compared to standard care. Prior to this work, there was no clear consensus regarding optimal lifestyle interventions for patients with early stage cancer. However, after performing direct comparisons of multiple dietary strategies, our research suggests that all reputable diets appear to be equally effective to achieve weight loss, BMI loss and reduced weight circumference. Additionally, combinations of diet and exercise appear to be associated with a larger probability of achieving a decrease in waist circumference when compared to dietary interventions alone. While body composition may be a more meaningful endpoint, the utility of this in routine oncological practice is limited. Larger studies incorporating interventions specifically designed to alter anthropometric measures and body composition with longer intervention periods and follow-up are warranted, to better define the role of lifestyle strategies in the management of patients with early stage cancer during and after treatment.

## Supporting information

S1 Text Systematic review protocol. (DOCX)

S2 Text Literature search strategy. (DOCX)

S3 Text Details of approach to network meta-analysis. (DOCX)

S4 Text Summary of eligible studies not included in NMAs. (DOCX)

S5 Text Study characteristics and risk of bias. (DOCX)

S6 Text NMA model fit statistics. (DOCX)

S7 Text League tables of NMA findings. (DOCX)

S8 Text Winbugs NMA code. (DOCX)

S9 Text PRISMA NMA checklist. (DOCX)

S1 Data Raw data, weight change. (XLSX)

S2 Data Raw data, BMI change. (XLSX)

S3 Data Raw data, waist circumference change. (XLSX)
