# Supplementary material for: Optimising weight-loss interventions in cancer patients—A systematic review and network meta-analysis
Source: PLoS One. 2021 Feb 4;16(2):e0245794. doi: 10.1371/journal.pone.0245794 (PMC7861370; doi:10.1371/journal.pone.0245794)
Supplement: S1 Text — (DOCX) [file pone.0245794.s001.docx]

**S1 Text:**

**Weight-control strategies in cancer patients -**

**Protocol for a systematic review and network meta-analysis**

***NOTE: The protocol for the current review was completed by team members from an oncology research unit and was not registered in the PROSPERO database prior to initiation. Provided below are the protocol details that were drafted to guide performance of the systematic review.***

**1. Introduction**

While obesity remains an under-recognized contributor to cancer incidence rates (1), up to 20% of cancer-related mortality are attributed to obesity (2-3). The World Health Organization defines overweight as a body mass index (BMI) between 25 and 29.9 kg/m^2^ and obesity as a BMI >30 kg/m^2^ (4). Accumulating evidence suggests that excessive energy intake and suboptimal levels of physical activity may be important after the diagnosis of cancer and influence the course of the disease, as well as overall health, well-being and survival (5). The importance of addressing obesity in cancer patients is shown by the recent ASCO position statement demonstrating their commitment to promoting research delineating the relationship between obesity and cancer, as well as educating oncologists in the current best practices for weight control in the oncology setting. Thus we have a paradox of some patients with cancer losing weight as a result of their disease and its treatment, yet at the global level obesity remains a preventable risk factor and important contributor to cancer mortality. While oncologists have not traditionally taken an active role in weight control management for their patients (1), they may not have perceived this effort as directly related to the treatment of cancer itself and may not appreciate the impact of obesity on cancer risk and implication with treatment. There are also few resources integrating the management of obesity in an oncology setting.

Obesity is a risk factor for poor wound healing, postoperative infections, lymphedema, and the development of comorbidities such as heart disease and diabetes, which all have a significant impact on cancer patients (1, 6). Although only investigated by a few studies, obesity may influence the efficacy of breast cancer treatment (7-8) by increasing estrogen and insulin growth factor-1 (IGF-1) production and through fat cell stimulation of the mammalian target of rapamycin. Obesity has further been linked to breast cancer recurrence (9-10) and decreased survival in pre- and post- menopausal breast cancer patients (11-12). Relationships between obesity and declined survivorship have also been established in ovarian (13), pancreatic (14), and endometrial cancer (15). The relationship between obesity and prostate cancer outcomes has also been extensively evaluated with a recent meta-analysis reporting a 20% increased risk of prostate-specific mortality in patients with an elevated BMI (16). Obese patients also have an increased risk of advanced disease at the time of diagnosis (17). While obesity may be associated with an increased risk of colon cancer recurrence and mortality, the evidence to support this is still lacking (18-19).

Many lifestyle intervention studies have been performed in cancer populations (20-22), which have demonstrated that lifestyle changes such as increased physical activity and improved dietary quality can lead to weight loss in survivors. One large-scale trial by Goodwin et al. (23) studied weight loss in 318 breast cancer patients who were randomized to different regimens of calorie restriction and exercise, while smaller studies (24-25) have assessed the benefits of weight-loss interventions such as dieticians, phone interventions, and exercise in cancer survivors. Several studies have evaluated the potential benefits of different forms of physical activity, standardized by MET output, in breast (26-29), colon (29), colorectal (30-32) and prostate (33-34) cancer patients and have demonstrated that exercise could be performed safely in adjuvant and post-treatment settings. Several studies have evaluated the potential benefits of dietary modifications (e.g. fat restriction, overall calorie restriction) after cancer diagnosis. The largest trial on dietary interventions by Chlebowski et al. (35) studied the potential benefits of dietary modifications on disease recurrence in women with early-stage breast cancer, concluding that a reduction in dietary fat intake may improve relapse-free survival of breast cancer patients. While smaller studies have looked at dietary interventions for weight loss in prostate cancer patients (36), larger studies are still needed.

While there are reviews on specific weight management interventions in the existing literature, there are currently no systematic reviews incorporating network meta-analyses attempting to identify optimal strategies for weight management for all types of cancers. The purpose of this systematic review is to explore and synthesize the available evidence evaluating weight control options in the management of cancer patients. In turn, this will allow for the identification of optimal intervention recommendations as well as determination gaps in the evidence that should be addressed by future clinical trials.

**Research Question of Interest to be Addressed in this Review?**

In clinical trials of cancer patients, is there a weight control strategy that has proven to be superior in terms of degree of weight control compared to others (defined as amount of body weight lost compared to baseline? In the context of this review, this will include named diets (Atkins, South Beach etc.), strict calorie restriction, exercise only or combined exercise/diet programs.

**Search Strategy**

In collaboration with a health information specialist, a detailed search strategy of EMBASE, Medline and the Cochrane library will be conducted based on the Population-Intervention-Comparator-Outcomes and Study framework (PICOS). The search will be limited to English language studies only. Stage 1 will include abstract screening to identify all potentially relevant citations. Stage 2 will include a full text review of relevant articles to determine which studies meet all inclusion criteria. Where applicable, authors will be contacted for unpublished data.

**Study Selection**

Once citations from the search are merged and duplicates are removed, two reviewers will first screen all titles and abstracts to identify potentially eligible RCTs for whom full text papers will be sought for further inspection (i.e. Stage 1 screening). Citations for which eligibility is unclear will also be carried forward to full text review (i.e. Stage 2 screening). Full texts will again be reviewed independently and in duplicate by the same pair of reviewers. Any disagreements regarding eligibility will be settled by consultation of a third party as needed. The process of study selection will be presented in the final report using a PRISMA flow diagram to highlight exclusions at all steps of the selection process.

**Study Selection Criteria**

**Population**:

- Human cancer patients over the age of 18.

**Intervention:**

- Weight control measures – diet, named diets (Mediterranean, Atkins, South Beach, etc), exercise, combination interventions
- Control arm either no weight control measure (standard care) or alternative weight control measure

**Outcomes**:

- Change in weight
- Change in BMI
- Change in waist circumference
- Progression free survival
- Overall survival

**Study design:**

- Randomized controlled trials
- Exclusion: Animal studies, non-randomized studies, publications in a language other than English, studies for the prevention of cancer in non-cancer populations

**Data requirements for outcome variables:**

**Change in weight, BMI and waist circumference:**

Intervention type, duration of intervention, on study monitoring, duration of follow up after intervention, absolute change in weight, if no individual patient data – median, average and ranges, absolute change in BMI.

**Progression free and Overall survival**

As above but with PFS and OS data. Cause of death, if documented (cancer related vs not). Hazard ratios and other summary measures of interest.

**Data Collection from Included Studies**

Data will be extracted from the relevant studies meeting the inclusion criteria using a standardized data collection form implemented in Microsoft Excel. The form will be piloted on a small number of studies and refined based on input from participating reviewers. All demographic measures (including age, gender, type of cancer and other measures) and outcomes will be collected with appropriate summary data, including means, standard deviations (for continuous outcomes), % values and #’s of events (for dichotomous outcomes) as needed and judged appropriate by data collectors. All included studies will be extracted independently by two reviewers. Disagreements will be resolved through discussion; if necessary, a third reviewer will be involved.

**Risk of bias assessment**

Full text articles will be independently assessed for methodological quality by two authors. The Cochrane Collaboration’s tool for assessing risk of bias in randomised trials will be used (35). The tool assesses for the following sources of bias; selection bias, performance / detection bias, attrition bias and reporting bias. Discrepancies in the initial independent assessments were resolved by discussion.

**Methods for Data Analysis**

The main objective of this review will be to derive comparisons between alternative strategies for weight control. If judged appropriate by the research team, groupings of interventions (e.g. by type of diet, type of exercise, etc) will be formed to form the basis for data analyses. We will work with the methods team from the Ottawa Hospital Methods Center and their statistical team to carry out Bayesian network meta-analyses to meet this objective. Biostatistical expertise and input will be sought prior to the initiation of data analysis to establish techniques and appropriate reporting methods for the final review.

**Reference List**

1. Ligibel JA, Alfano CM, Courneya KS, et al. American Society of Clinical Oncology Position Statement on Obesity and Cancer. J Clin Oncol 2014; 32(31): 3568-3574
2. National Cancer Institute NC: Fact sheet: Obesity and cancer risk. <http://www.cancer.gov/cancertopics/factsheet/risk/obesity>
3. Protani M, Coory M, Martin JH. Effect of obesity on survival of women with breast cancer: systematic review and meta-analysis. Breast Cancer Res Treat 2010; 123(3):627-635.
4. Shah NR, Braverman ER: Measuring adiposity in patients: The utility of body mass index (BMI), percent body fat, and leptin. PLoS One 2012;7:e33308
5. Denmark-Wahnefried, Platz EA, Ligibel, et al. The role of obesity in cancer survival and recurrence. Cancer Epidemiology, biomarkers and prevention 2012; 21(8).
6. Guh DP, Zhang W, Bansback N, et al. The incidence of co-morbidities related to obesity and overweight: a systematic review and meta-analysis. BMC Public Health 2009; 9(88).
7. Pfeiler G, Konigsberg R, Fesl C, et al. Impact of body mass index on the efficacy of endocrine therapy in premenopausal patients with breast cancer: an analysis of the prospective ABCSG-12 Trial. J Clin Oncol. 2011;29(19):2653–2659.
8. de Azambuja E, McCaskill-Stevens W, Francis P, et al. The effect of body mass index on overall and disease-free survival in node-positive breast cancer patients treated with docetaxel and doxorubicin-containing adjuvant chemotherapy: the experience of the BIG 02-98 trial. Breast Cancer Res Treat 2010;119(1):145–153.
9. Ligibel J. Obesity and breast cancer. Oncology (Williston Park) 2011; 25:994–1000.
10. Druesne-Pecollo N, Touvier M, Barrandon E, et al. Excess body weight and second primary cancer risk after breast cancer: a systematic review and meta-analysis of prospective studies. Breast Cancer Research and Treatment 2012; 135(3):647-654
11. Chan DA, Vieira AR, Aune D, et al. Body mass index and survival in women with breast cancer-systematic literature review and meta-analysis of 82 follow-up studies. [Ann Oncol.](http://www.ncbi.nlm.nih.gov/pubmed/?term=Chan+D%2C+Body+mass+index+and+survival+in+women+with) 2014 Oct;25(10):1901-14.
12. Protani M, Coory M, Martin JH. Effect of obesity on survival of women with breast cancer: systematic review and meta-analysis. Breast Cancer Res Treat 2010;123: 627–635.
13. Protani MM, Nagle CM, Webb PM. Obesity and ovarian cancer survival: a systematic review and meta-analysis. Cancer Prev Res 2012;5(7):901-910.
14. Li D, Morris JS, Liu J, et al. Body mass index and risk, age of onset, and survival in patients with pancreatic cancer.JAMA 2009; 301:2553–2562
15. Arem H, Irwin ML. Obesity and endometrial cancer survival: a systematic review. Int J Obes 2013; 37(5):634-9.
16. Cao Y, Ma J. Body mass index, prostate cancer-specific mortality, and biochemical recurrence: a systematic review and meta-analysis. Cancer Prev Res 4:486-501
17. [Discacciati A](http://www.ncbi.nlm.nih.gov/pubmed/?term=Discacciati%20A%5BAuthor%5D&cauthor=true&cauthor_uid=22228452)^1^, [Orsini N](http://www.ncbi.nlm.nih.gov/pubmed/?term=Orsini%20N%5BAuthor%5D&cauthor=true&cauthor_uid=22228452), [Wolk A](http://www.ncbi.nlm.nih.gov/pubmed/?term=Wolk%20A%5BAuthor%5D&cauthor=true&cauthor_uid=22228452). Body mass index and incidence of localized and advanced prostate cancer--a dose-response meta-analysis of prospective studies. [Ann Oncol.](http://www.ncbi.nlm.nih.gov/pubmed/22228452) 2012 Jul;23(7):1665-71.
18. Meyerhardt JA, Catalano PJ, Haller DG, et al: Influence of body mass index on outcomes and treatment-related toxicity in patients with colon carcinoma. Cancer 2003; 98:484-495.
19. Dignam JJ, Polite BN, Yothers G, et al: Body mass index and outcomes in patients who receive adjuvant chemotherapy for colon cancer. J Natl Cancer Inst 2006; 98:1647-1654.
20. Schmitz KH, Courneya KS, Matthews C, et al: American College of Sports Medicine roundtable on exercise guidelines for cancer survivors. Med Sci Sports Exerc 2010;42:1409-1426
21. Ligibel J: Obesity and breast cancer. Oncology (Williston Park) 2011; 25:994-1000
22. Galvão DA, Newton RU: Review of exercise interventions studies in cancer patients. J Clin Oncol 2005; 23:899-909
23. [Goodwin PJ](http://www.ncbi.nlm.nih.gov/pubmed?term=Goodwin%20PJ%5BAuthor%5D&cauthor=true&cauthor_uid=24934783)^1^, [Segal RJ](http://www.ncbi.nlm.nih.gov/pubmed?term=Segal%20RJ%5BAuthor%5D&cauthor=true&cauthor_uid=24934783)^2^, [Vallis M](http://www.ncbi.nlm.nih.gov/pubmed?term=Vallis%20M%5BAuthor%5D&cauthor=true&cauthor_uid=24934783)^2^, et al. Randomized trial of a telephone-based weight loss intervention in postmenopausal women with breast cancer receiving letrozole: the LISA trial. [J Clin Oncol.](http://www.ncbi.nlm.nih.gov/pubmed/24934783) 2014 Jul 20;32(21):2231-9. doi: 10.1200/JCO.2013.53.1517. Epub 2014 Jun 16.
24. Playdon M, Thomas G, Sanft T, et al. Weight loss intervention for breast cancer survivors: A systematic review. Current Breast Cancer Reports 2013; 5(3):222-246
25. Demark-Wahnefried W, Campbell KL, Hayes SC. Weight management and its role in breast cancer rehabilitation. Cancer 2012; 111(S8)
26. Holmes M, Chen W, Feskanich D, et al. Physical activity and survival after breast cancer diagnosis. JAMA 2005; 293:2479-2486
27. Holick C, Newcomb P, Trentham-Dietz A, et al. Physical activity and survival after diagnosis of invasive breast cancer. Cancer epidemiology, Biomarkers and prevention 2008; 17:379-386
28. Sternfeld B, Weltzien E, Quesenberry CP, et al. Physical activity and risk of recurrence and mortality in breast cancer survivors: findings from the LACE study. Cancer Epidemiol Biomarkers Prev 2009; 18:87-95
29. Courneya KS, Mackey JR, Bell GJ, Jones LW, Field CJ, Fairey AS. Randomized controlled trial of exercise training in postmenopausal breast cancer survivors: cardiopulmonary and quality of life outcomes.J Clin Oncol 2003;21:1660 – 8.
30. Meyerhardt J, Heseltine D, Niedzweicki D, et al. Impact of physical activity on cancer recurrence and survival in patients with stage III colon cancer: Findings from CALGB 89803. JCO 2006; 22:3535-3541
31. Meyerhardt JA, Giovannucci EL, Ogino S, et al. Physical activity and male colorectal cancer survival. Arch Intern Med 2009; 169:2102-8
32. Courneya KS, Friedenreich CM, Quinney HA, Fields AL, Jones LW, Fairey AS. A randomized trial of exercise and quality of life in colorectal cancer survivors. Eur J Cancer Care (Engl) 2003;12:347 – 57.
33. Meyerhardt J, Giovannucci E, Holmes M, et al. Physical activity and survival after colorectal cancer diagnosis. JCO 2006; 24:3527-3534
34. Richman EL, Kenfield SA, Stampfer MJ et al. Physical activity after diagnosis and risk of prostate cancer progression: data from the cancer of the prostate strategic urologic research endeavor. Cancer Res 71:3889-95
35. [Chlebowski RT](http://www.ncbi.nlm.nih.gov/pubmed?term=Chlebowski%20RT%5BAuthor%5D&cauthor=true&cauthor_uid=17179478)^1^, [Blackburn GL](http://www.ncbi.nlm.nih.gov/pubmed?term=Blackburn%20GL%5BAuthor%5D&cauthor=true&cauthor_uid=17179478), [Thomson CA](http://www.ncbi.nlm.nih.gov/pubmed?term=Thomson%20CA%5BAuthor%5D&cauthor=true&cauthor_uid=17179478), et al. Dietary fat reduction and breast cancer outcome: interim efficacy results from the Women's Intervention Nutrition Study. J Natl Cancer Inst 2006; 98(24): 1767-76
36. Van Patten CL, de Boer JG, Tomlinson GES. Diet and dietary supplement intervention trials for the prevention of prostate cancer recurrence: a review of the randomized controlled trial evidence. J Urol 2008; 180:2314-21
