# Supplementary material for: Optimising weight-loss interventions in cancer patients—A systematic review and network meta-analysis
Source: PLoS One. 2021 Feb 4;16(2):e0245794. doi: 10.1371/journal.pone.0245794 (PMC7861370; doi:10.1371/journal.pone.0245794)
Supplement: S2 Text — (DOCX) [file pone.0245794.s002.docx]

**S2 Text: Medline Search Strategy**

Database: Ovid MEDLINE(R) In-Process & Other Non-Indexed Citations and Ovid MEDLINE(R) <1946 to Present>

--------------------------------------------------------------------------------

1 exp Neoplasms/

2 exp Antineoplastic Agents/

3 combined modality therapy/ or chemotherapy, adjuvant/ or neoadjuvant therapy/

4 exp Radiotherapy/

5 exp Surgical Procedures, Operative/

6 Survivors/

7 or/2-6

8 1 and 7

9 ((cancer or neoplasm$) adj5 (patient$ or surviv$ or treatment or therapy or surg$ or resect$

or radiotherap$ or radiation therap$ or irradiation or chemotherap$)).tw. (504735)

10 exp Neoplasms/dt, rt, su, th

11 or/8-10

12 Weight Reduction Programs/

13 exp Diet Therapy/

14 *weight loss/

15 Energy Intake/

16 weight management.tw.

17 ((weight loss or weight reduc$) adj3 (counsel$ or program$ or intervention$ or therap$)).tw.

18 (calor$ adj2 restrict$).tw.

19 ((mediterranean or atkins or south beach) adj2 diet$).tw.

20 weight watcher$.tw.

21 (diet$ adj3 (counsel$ or therap$ or management or intervention$)).tw.

22 exp Exercise/ or exercise therapy/

23 (exercis$ or physical activit$).tw.

24 or/12-23

25 11 and 24

26 randomized controlled trial.pt.

27 controlled clinical trial.pt.

28 random$.tw.

29 placebo.ab.

30 clinical trials as topic/

31 trial.ti.

32 or/26-31

33 animals/ not humans/

34 32 not 33

35 25 and 34

36 (201505* or 201506* or 201507* or 201508* or 201509* or 20151* or 2016*).dc.

37 35 and 36

38 limit 37 to english language
