# Supplementary material for: Optimising weight-loss interventions in cancer patients—A systematic review and network meta-analysis
Source: PLoS One. 2021 Feb 4;16(2):e0245794. doi: 10.1371/journal.pone.0245794 (PMC7861370; doi:10.1371/journal.pone.0245794)
Supplement: S3 Text — (DOCX) [file pone.0245794.s003.docx]

**S3 Text: Details of Approach to Network Meta-Analysis**

This file is provided to present a more detailed version of the approach detailed in the main text for readers seeking additional detail regarding our approach to model fitting and evaluation for the analyses presented in the review.

**Overview of Modeling Approach**

NMA is an extension of traditional pairwise meta-analysis which enables the comparison of multiple interventions in a single analysis, and which allows for incorporation of both direct and indirect evidence of relevance. NMAs of the changes from baseline in body weight, body mass index and waist circumference were performed. The nature of reporting these endpoints varied across included studies, with some reporting changes from baseline while others reported mean values of each endpoint at baseline and follow-up, with standard deviations for each. For the latter, we calculated the mean changes from baseline and imputed the standard errors of the mean changes according to, respectively, where and are the standard deviations of the endpoints at baseline and at follow-up, and is the sample size of the arm in the study. The correlation between the outcomes at baseline and follow-up was assigned a Uniform prior distribution, . We adapted the fixed effects (FE) and random effects (RE), three-level hierarchical models with a Normal likelihood and identity link which are based on the mean changes from baseline and corresponding standard errors, with clustering of the interventions into 4 groups (standard care, diet interventions, exercise interventions and combined interventions); the main text focuses upon class level comparisons, while the intervention level comparisons are reported in detail in the report appendices. The median values of estimated mean differences (MD) of interventions versus standard care were reported along with corresponding 95% credible intervals. Forest plots are presented to summarize findings versus the standard care group, while all possible pairwise comparisons between groups are summarized using league tables provided in this review’s online supplement. Selection between FE and RE models was based upon comparison of deviance information criteria (DIC) between models, with a threshold of five points or more suggesting an important difference in model fit; fit statistics are also reported in the review supplement. The assumption of consistency between direct and indirect evidence was assessed by plotting the posterior mean deviance contributions from the consistency model against those from the unrelated means model to see if they aligned. All NMAs were performed using OpenBUGS software version 3.2.3 and the R package R2OpenBUGS. Model convergence was assessed using established methods including Gelman-Rubin diagnostics and the Potential Scale Reduction Factor. Findings reported within the main text of the review focus upon results from NMAs, while author conclusions of the remaining studies that did not appropriately fit into the NMAs (due to the types of comparisons made or lack of sufficient data) are summarized in the appendices. The Comparison-adjusted funnel plots were applied to assess for small-study effects as signals of publication bias.
