# Supplementary material for: Optimising weight-loss interventions in cancer patients—A systematic review and network meta-analysis
Source: PLoS One. 2021 Feb 4;16(2):e0245794. doi: 10.1371/journal.pone.0245794 (PMC7861370; doi:10.1371/journal.pone.0245794)
Supplement: S4 Text — (DOCX) [file pone.0245794.s004.docx]

**S4 Text: List of eligible studies not included in NMAs**

In total, 75 of the 98 included articles had data that could be incorporated into the NMAs presented in this review. Below, we provide a description of the reasons for which the remaining 23 studies were not included in these analyses. For reasons related to network structure, the research was not planned to consider differences in method of delivery, intensity, or the granular details of dietary and exercise interventions etc. Behavioral therapies were not considered of interest for the NMAs. Pragmatic pooling of data into classes was performed in order to translate the findings of this study to clinical practice and “standard care” arms were grouped together. Any studies with an intervention arm or control arm which did not align with the structure of the network were not included in NMAs. Study description, endpoints reported, relevant findings and reasons for exclusion are outlined below.

| Author; year (ref) | Study description | Endpoints | | | Summary of Findings | Reason for exclusion from NMA |
| --- | --- | --- | --- | --- | --- | --- |
|  | | BMI | WT | WC |  |  |
| Lahart et al.; 2018 (122) | Single center RCT comparing home-based exercise with counselling to usual care. | X |  |  | The home-based physical activity intervention led to increases in cardiorespiratory fitness and self-reported physical activity, although no other beneficial improvements were noted for the other outcomes of interest, including BMI. | Focused on the effect of counselling. Comparison of different types of exercise which were not included in the network. |
| Artene et al.; 2017 (52) | Single center RCT comparing a high protein, calcium, probiotics and prebiotics diet to this diet and isometric exercises. |  | X |  | Weight loss and fat loss was seen in both arms, with more pronounced losses when diet was combined with exercise. | Comparison of an intervention which was not included in the network. |
| Kwiatkowski et al.; 2017 (109) | Multi center RCT comparing daily supervised physical training, dietary education, physiotherapy and psychological support to consultations with a dietician every 6 months for 3 years. |  | X | X | Increase in QOL and weight control and reductions in waist circumference were found at 2 years. | Only percentage of change in WT and WC were reported. |
| Ghavami et al.; 2017 (113) | Single center RCT comparing dietary energy-restriction training and aerobic exercises to usual care. | X |  |  | Significant improvements in fatigue, BMI and QOL as well as quality of sleep were found in the intervention arm. | Comparison of an intervention which was not included in the network (included patient individualization). |
| Stolley et al.; 2017 (123) | Multi center RCT comparing interventionist-guided weight loss program to self-guided weight loss program. |  | X | X | Weight loss and body composition changes were greater in the interventionist-guided versus self-guided groups at 6 and 12 months and a greater number of participants achieved the target of 5% weight loss. | Interventions compared method of delivery. |
| Sedjo et al.; 2016 (124) | Multi center RCT comparing active goal setting of weight, dietary intake and physical activity to individualized weight loss counselling. |  | X |  | Patients in the intervention arm had fewer new medical conditions. Fewer new medical conditions were associated with greater weight loss. | Focused on behavioural therapy. |
| Devin et al.; 2015 (125) | Single center RCT comparing high-intensity aerobic exercise to moderate-intensity aerobic exercise. |  | X |  | High-intensity was superior to moderate-intensity aerobic exercise to improve VO2 peak and lead to significant increases in lean mass, decreases in fat mass and fat percentage. | Comparison of different exercise intensities. |
| Greenlee et al.; 2015 (99) | Single center RCT comparing phyto rich, low-fat diet to standard reading material. | X | X | X | Higher increases in fruit and vegetable intake at 12 months. No significant decrease in weight, BMI or waist circumference. | Data used for NMA from related publication by Greenlee et al. (2016) (115). |
| Anderson et al.; 2016 (126) | Single center RCT comparing low-calorie, low-fat, aerobic exercise to usual care. |  | X | X | Significant improvements in health eating index (HEI) were achieved with the intervention arm. Those with weight loss >=5% of body weight had significantly greater improvements in HEI score. | Data used for NMA from related publication by Harrigan et al. (2016) (91). |
| Toohey et al.; 2015 (127) | Single center RCT comparing low-volume high-intensity (LVHIIT) aerobic physical exercise to continuous low to moderate intensity aerobic physical exercise. |  | X | X | Significantly greater weight loss was observed with the LVHI intervention arm compared to the control arm. No effect was noted on waist-circumference. | Comparison of different exercise intensities. |
| Winters-Stone et al.; 2015 (128) | Single center RCT comparing resistance and impact exercises to whole-body stretching and relaxation control. |  | X |  | Significant decreases in weight and fat mass were achieved in the intervention arm as compared to weight gain and increased body fat in the control arm. | Comparison of a control group which was not included in the network. |
| Courneya et al.; 2015 (129) | Single center, crossover, RCT comparing aerobic exercise to wait-list control. |  |  |  | No significant differences in 5-year PFS were noted in the ITT population. | Reported on OS/DFS outcomes. |
| Galvao et al.; 2014 (130) | Multi center RCT comparing supervised aerobic and resistance exercise to a self-directed aerobic program with a pedometer. |  | X | X | Participants undergoing supervised exercise showed improvement in cardiorespiratory fitness at 6 and 12 months. No significant differences in weight and waist circumference were found. | Interventions compared method of delivery. |
| Trinh et al.; 2014 (131) | Single center RCT comparing aerobic exercise with exercise counselling to behavioural counselling. | X | X | X | No significant difference in self-reported physical activity at 12 weeks was noted. No significant differences in anthropometric measures were found. | Comparison regarding methods of delivery/co-intervention with aerobic exercise (exercise counselling to behavioural counselling). |
| Hawkes et al.; 2013 (132) | Multi center RCT comparing health coaching on exercise and diet to usual care. | X |  |  | Significant intervention effects observed for moderate physical activity, BMI and dietary habits at 12 months with health coaching. | Comparison of health coaching for lifestyle intervention delivery. |
| Hebert et al.; 2012 (133) | Multi center cross over RCT comparing diet and exercise counselling to standard care. | X |  |  | No differences were observed between the intervention arm and control arm for PSA change or BMI. | Comparison of counselling for lifestyle intervention delivery. |
| Littman et al.; 2012 (134) | Single center, cross over RCT comparing a combined supervised and home-based yoga program to wait-list control. | X | X | X | QOL and fatigue improved in the intervention arm. Changes in waist circumference were greater in the intervention arm; no significant changes in weight or BMI were found. | Comparison of different types of exercise which were not included in the network. |
| Winters-Stone et al.; 2011 (135) | Multi center RCT comparing supervised aerobic and resistance exercise to flexibility and relaxation. |  | X |  | Resistance and impact exercise was associated with preservation of BMD and increases in lean mass. Weight change was not significantly different. | Comparison of a control group which was no included in the network. |
| Djuric et al.; 2011 (136) | Single center RCT comparing aerobic exercise and fruit/vegetable rich low-calorie diet via written materials compared to telephone counseling. | X | X | X | Mean body fat and waist circumference decreased in the intervention arm using telephone counselling. No significant differences in weight and BMI were found. | Comparison of methods of delivery (written materials to telephone counselling). |
| Demark-Wahnefried et al.; 2008 (137) | Multi-centre RCT comparing a low-fat and flaxseed diet to flaxseed only or standard of care. | X |  |  | Lower levels of proliferation were found for the patients assigned to flaxseed arms. No differences in BMI were found in the ITT analyses, although these were significant in the PP analyses. | Comparison of an alternate dietary interventions which were not included in the network. |
| Li et al.; 2008 (138) | Single-center RCT comparing a low-fat, high-fiber soy diet to standard of care. | X | X |  | Significant differences in dietary fat and increase in fiber was noted. No significant differences in weight or BMI were found. | Comparison of an alternate dietary intervention which was not included in the network. |
| Carmack et al.; 2006 (139) | Single center RCT comparing a group-based lifestyle physical activity program to group-based educational support program and a standard of care program. | X |  | X | No significant changes in QOL or anthropometric measures were found. | Comparison of counselling to an educational exercise intervention. |
| Ohira et al.; 2006 (140) | Single center, crossover RCT comparing resistance/aerobic exercise to wait-list control. | X | X | X | QOL was significantly better in the intervention group. Increases in lean mass were correlated with improvements in physical and psychosocial global scores. | Data used for NMA from related publication by Schmitz et al. (2005) (79) |
| Loprinzi et al.; 1996 (141) | Single center RCT comparing dietician counselling for weight maintenance to standard care. |  | X |  | Less weight gain was noted in the intervention arm numerically, which was not statistically significant. | Comparison counselling. |
| DeWaard et al.; 1993 (142) | Multi center cross over RCT comparing low-calorie diet to standard care. |  | X |  | Greater weight loss was achieved in the intervention arm. | Baseline values not reported. |
