# Supplementary material for: Optimising weight-loss interventions in cancer patients—A systematic review and network meta-analysis
Source: PLoS One. 2021 Feb 4;16(2):e0245794. doi: 10.1371/journal.pone.0245794 (PMC7861370; doi:10.1371/journal.pone.0245794)
Supplement: S5 Text — (DOCX) [file pone.0245794.s005.docx]

**S5 Text: Study Characteristic Tables of Studies Included in NMA**

|  | **Overview of Study Characteristics** | | | | | | | | | | | | | |
| --- | --- | --- | --- | --- | --- | --- | --- | --- | --- | --- | --- | --- | --- | --- |
| **Primary Study and Patient Characteristics** | | | | | | | **Intervention Details** | | | | | **Endpoints reported** | | |
| **Author; year (ref)** | | **Design details; study/**  **FU durations** | **Intervention Type** | **Mean age (years)** | **Cancer Types Enrolled** | **# of patients** | **Group 1** | **Control** | **Groups 2 and 3 (where present)** | **Efficacy vs effectiveness** | **Time of Intervention** | **BMI** | **WT** | **WC** |
| Freedland et al.; 2019 (45) | | MC, RCT; 6 mo / 6 mo | Combination | 66 | Prostate | 29 | Low-carbohydrate <=20g/day and walking >30 min >= 5 days/week | Usual care |  | Efficacy | During treatment (ADT) | Y | Y |  |
| McNeil et al.; 2019(46) | | SC, RCT; 12 wks / 24 wks | Exercise | 59 | Breast | 45 | 300 min/wk moderate intensity aerobic exercise (40-59% HR reserve) | Usual care | 150 min/wk high intensity aerobic (60-80% HR reserve) | Effectiveness | Post treatment | Y |  | Y |
| Mijwel et al.; 2019(47) | | MC, RCT; 16 wks / 104 wks | Exercise | 53 | Breast | 179 | High-intensity aerobic interval training with resistance training | Usual care | Moderate-intensity aerobic training | Efficacy | During treatment | Y | Y |  |
| Bourke et al.; 2018(48) | | MC, RCT; 12mo / 12mo | Exercise | 67 | Prostate | 50 | 140min/wk aerobic + 2 group sessions/wk at 65-85% maximal predicted HR | Usual care |  | Efficacy | Post treatment (Active surveillance) | Y | Y |  |
| Dieli-Conwright et al.; 2018 (49) | | MC, RCT; 16 wks / 16 wks | Exercise | 54 | Breast | 21 | Supervised aerobic and resistance exercise 3x/wk | Wait-list control |  | Efficacy | Post treatment |  | Y | Y |
| Henning et al.; 2018(50) | | MC, RCT; 5-8 wks / 8 wks | Combination | 62 | Prostate | 34 | Restriction 1200-1500kcal/d with 1h/d aerobic, resistance exercise | Usual care |  | Efficacy | During treatment (awaiting prostatectomy) | Y | Y | Y |
| Toohey et al.; 2018(51) | | MC, RCT; 12 wks / 12 wks | Exercise | 51 | Breast, ovarian, appendix, anal, cervical, liver, esophageal, melanoma, leiomyoma, unknown primary | 75 | Low-volume high intensity interval training | Usual care | Continuous low to moderate intensity exercise training | Efficacy | Post treatment (within 24mo diagnosis) |  | Y | Y |
| Braakhuis et al.; 2017(53) | | MC, RCT; 6 mo / 6 mo | Dietary | 55 | Breast | 50 | Mediterranean diet | Usual care | Low-fat diet (based on the New Zealand Ministry of Health Eating and Activity Guideslines for Adults | Efficacy | Post treatment | Y | Y | Y |
| Demark-Wahnefried et al.; 2017(54) | | SC, RCT; average 50d / average 50d | Combination | 60 | Prostate | 40 | Low-calorie deficit 1000kcal/d, 60-80% maximal heart rate with aerobic exercise | Wait-list control |  | Efficacy | During treatment (awaiting prostatectomy) | Y | Y |  |
| Irwin et al.; 2017(55) | | MC, RCT; 12 wks / 12 wks | Exercise | 59 | Breast, colon, rectum, lung, endometrial, prostate, lymphoma, other | 186 | 90 min 2x/wk per ACSM and YMCA program | Wait-list control |  | Efficacy | Post treatment | Y |  |  |
| Skouroliakou et al; 2018 (106) | | SC, RCT; 6 mo / 6mo | Combination | - | Breast | 70 | Personalized diet (based on Mediterranean diet) |  | Updated American  Cancer Society Guidelines on Nutrition and Physical  Activity for Cancer Prevention and ad libitum diet | Effectiveness | Post treatment | Y | Y | Y |
| Arikawa et al; 2017 (105) | | SC, RCT; 12 wks/6 wks | Combination | 57 | Breast | 21 | Calorie-restricted feeding plus exercise |  | Weight management counseling | Efficacy | Post treatment |  | Y |  |
| Wall et al; 2017 (107) | | SC, RCT; 6 months | Exercise | 69 | Prostate | 97 | Combined aerobic and resistance  training program | Usual care |  | Efficacy | During treatment (ADT) |  | Y |  |
| Roveda et al; 2017 (108) | | SC, RCT; 3 months | Exercise | 57 | Breast | 40 | Active PA program that included 2 sessions of 1-hour brisk  walking per week + WCRF/AICR recommendation |  | WCRF/AICR recommendation  to be physically active | Efficacy | Post treatment | Y | Y | Y |
| Thomas et al; 2017 (110) | | SC, RCT; 12 months | Exercise | 62 | Breast | 121 | Aerobic and resistance exercise | Usual  care |  | Efficacy | Post treatment | Y | Y |  |
| Hojan et al; 2017 (111) | | SC, RCT; 12 months | Exercise | 66 | Prostate | 72 | Individually tailored exercise  program (physical exercise group trained 5 d/  wk during RT and then 3 d/wk) | Usual care |  | Efficacy | During treatment (ADT) | Y | Y |  |
| Kim et al; 2017 (112) | | SC, RCT; 12 wks | Exercise | 52 | Breast | 30 | Exercise training program (following the American College of Sports Medicine's  guide to exercise and cancer survivorship) | Usual care |  | Efficacy | Post treatment |  | Y | Y |
| Rogers et al; 2016 (114) | | MC, RCT; 3 months/3 months | Exercise | 54 | Breast | 222 | Social cognitive theory based  Better Exercise Adherence after Treatment  for Cancer | Usual care |  | Efficacy | Post treatment | Y |  |  |
| Greenlee et al; 2016 (115) | | SC, RCT; 12 wks/ 9 months | Diet | 56 | Breast | 70 | Short-term culturally based  dietary intervention on increasing fruits/vegetables, decreasing  fat | Standard of care written dietary recommendations for  cancer survivors |  | Efficacy | Post treatment | Y | Y | Y |
| Cantarero-Villaneuva et al; 2016 (116) | | SC, RCT; 8 wks/ 4 months | Exercise | 60 | Colon | 46 | Trunk muscle stabilization exercise | Usual-care |  | Efficacy | Post treatment | Y | Y | Y |
| Befort et al; 2016 (117) | | MC, RCT; phase1: 6 months | Combination | 58   \| \|  \| \| --- \| \| \| --- \| --- \| | Breast | 210 (phase 1) | Group conference call training; diet + PA |  | Mail-based recommendation on diet and PA | Efficacy | Post treatment |  | Y |  |
| Winters-Stone et al; 2016 (118) | | SC, RCT; 6 months | Exercise | 72 | Prostate | 64 couples | Partnered  strength training program | Usual care |  | Efficacy | Post treatment |  | Y |  |
| Reeves et al; 2016 (119) | | SC, RCT; 6 months | Combination | 55 | Breast | 90 | Telephone-delivered diet and physical  activity advice | Usual care |  | Effectiveness | Post treatment |  | Y | Y |
| Courneya et al; 2016 (120) | | MC, RCT; 3 years | Combination | 60 | Colon | 273 | Structured exercise program recreational aerobic/PA |  | Health education promoting PA and healthy nutrition | Efficacy | Post treatment |  | Y | Y |
| Brown et al; 2017 (121) | | SC, RCT; 15 weeks | Combination | 56 | Breast cancer, and other solid tumors and hematologic malignancies | 60 | Clinic-based weight loss intervention; Cal-restriction + PA | Usual care |  | Effectiveness | Post treatment |  | Y |  |
| Dolan et al; 2016 (89) | | SC, RCT; 6 wks/6 wks | Exercise | 57 | Breast | 33 | Continuous aerobic | Standard care | Interval-training aerobic | Efficacy (supervised) | Post treatment |  | Y | Y |
| Gilbert et al; 2016 (90) | | SC, RCT; 12 wks/24 wks | Combination | 70 | Prostate | 50 | Aerobic + resistance + low-fat / low-carb | Standard care |  | Efficacy (supervised) | During treatment (ADT) | Y |  |  |
| Harrigan et al; 2016 (91) | | MC, RCT; 6 mo / 6 mo | Combination | 59 | Breast | 100 | In-person low-cal, low-fat, aerobic | Standard care | Telephone low-calorie, low-fat, aerobic | Efficacy vs effectiveness (in-person vs phone) | Post treatment |  | Y | Y |
| Hvid et al; 2016 (92) | | SC, RCT; 24 mo/ 24 mo | Exercise | 69 | Prostate | 25 | Aerobic exercise | Standard care |  | Effectiveness (home based) | During treatment (ADT), active surveillance | Y | Y |  |
| Lahart et al; 2016 (122) | | SC, RCT; 6 mo /6 mo | Exercise | 54 | Breast | 80 | Aerobic exercise | Standard care |  | Effectiveness (mailed material) | Post treatment | Y | Y |  |
| Sheppard et al; 2016 (94) | | MC, RCT; 12 wks/12wks | Combination | 54 | Breast | 22 | Aerobic exercise + low-fat / FV diet | Standard care |  | Efficacy  (direct contact) | Post treatment | Y | Y | Y |
| Brown et al; 2015 (95) | | MC, CO, RCT; 12 mo/ 12mo | Exercise | 56 | Breast | 294 | Resistance | Standard care |  | Efficacy (trainers, logs) | Post treatment | Y | Y |  |
| Casla et al; 2015 (96) | | MC, RCT; 12 wks/12 wks | Combination | 49 | Breast | 94 | Aerobic + resistance, Mediterranean diet | Standard care |  | Efficacy (supervised + counselling) | Post treatment | Y | Y |  |
| Cornette et al; 2015 (97) | | SC, RCT; 27 wks / 54 wks | Exercise | 51 | Breast | 42 | Aerobic + resistance | Standard care |  | Effectiveness (home-based) | Post treatment | Y |  |  |
| Edvardsen et al; 2015 (98) | | SC, RCT; 20 wks / 24 wks | Exercise | 65 | Lung | 61 | Aerobic + resistance training | Standard care |  | Efficacy (trainer, logs) | Post treatment | Y |  |  |
| Kampshoff et al; 2015 (100) | | MC, CO, RCT; 12 wks/12wks | Exercise | 54 | Mixed (Breast, colon, ovarian, lymphoma, cervix, testis) | 277 | High-intensity aerobic, resistance | Wait-list | Low-moderate intensity aerobic, resistance | Efficacy (physiotherapist) | Post treatment | Y |  |  |
| Nilsen et al; 2015 (101) | | MC, RCT; 16 wks/16wks | Exercise | 66 | Prostate | 58 | Strength training | Standard care |  | Efficacy (supervised) | During treatment (ADT) | Y | Y |  |
| Swisher et al; 2015 (102) | | MC, RCT; 12 wks/12wks | Combination | 54 | Breast | 28 | Aerobic + / -resistance, low-cal | Standard care |  | Efficacy (supervised) | Post treatment | Y | Y | Y |
| Travier et al; 2015 (103) | | MC, CO, RCT; 18 wks/36 wks | Exercise | 50 | Breast | 204 | Aerobic + resistance | Standard care |  | Efficacy (supervised) | During treatment |  | Y |  |
| Van Vulpen et al; 2015 (104) | | MC, CO, RCT; 18 wks/36 wks | Exercise | 58 | Colorectal | 33 | Aerobic + resistance | Standard care |  | Efficacy (supervised) | During treatment |  | Y |  |
| Cormie et al; 2015 (83) | | SC, RCT; 3 mo / 3 mo | Exercise | 68 | Prostate | 63 | Aerobic + resistance | Standard care |  | Effectiveness (supervised) | During treatment (ADT) |  | Y |  |
| Backman et al; 2014 (60) | | MC, RCT; 10 wks/ 10 wks | Exercise | 54 | Breast, colorectal | 67 | Aerobic | Standard care |  | Effectiveness (unsupervised) | During treatment | Y | Y | Y |
| Cho et al.; 2014 (56) | | SC RCT; 8 wks / 9 mo | Dietary | 46 | Breast | 61 | Phytochemical rich diet | Standard care |  | Effectiveness (nutrition counselling, cooking class) | Post treatment | Y | Y | Y |
| Courneya et al.; 2014 (129) | | MC, CO, RCT ; 6 mo/ 89 mo | Exercise | 50 | Breast | 242 | Aerobic | Standard care | Resistance | Efficacy (supervised exercise program) | During treatment |  | Y |  |
| Demark-Wahnefried et al.; 2014 (58) | | MC RCT; 12 mo/ 12 mo | Combination | 61 | Breast | 136 | Resistance / aerobic exercise + low-calorie diet | Standard care | Resistance/ aerobic exercise + low-calorie diet group | Effectiveness (tailored print intervention) | Post treatment | Y | Y | Y |
| Goodwin et al.; 2014 (59) | | MC RCT; 36 mo / 24 mo | Combination | 61 | Breast | 338 | Aerobic exercise + low-calorie diet | Standard care |  | Effectiveness (phone based intervention) | During treatment (hormone therapy) |  | Y |  |
| Greenlee et al.; 2013 (61) | | SC, CO, RCT; 6 mo / 12 mo | Combination | 51 | Breast | 42 | Curves program (exercise circuit, low-fat / low-cal diet) | Wait-list |  | Efficacy (supervised Curves program) | Post treatment |  | Y | Y |
| Scott et al.; 2013 (62) | | SC RCT; 24 wks / 24 wks | Combination | 56 | Breast | 90 | Resistance / aerobic exercise + low-calorie diet | Standard care |  | Efficacy (supervised exercise and tailored diet) | Post treatment | Y | Y | N |
| Wright et al; 2013 (84) | | MC, RCT; 6 wks / 6 wks | Dietary | 58 | Prostate | 19 | Low-fat, low-cal | Standard care |  | Effectiveness (self-help program) | Post treatment | Y | Y |  |
| Demark-Wahnefried et al.; 2012 (32) | | MC, CO, RCT; 12 mo / 24 mo | Combination | 73 | Breast, colorectal, prostate | 488 | Resistance / aerobic exercise + low-fat / phyto rich diet | Wait-list |  | Efficacy (mailed print and phone counselling) | Long-term post treatment | Y | Y |  |
| Oneill et al; 2012 (85) | | SC, CO, RCT; 6 mo / 6 mo | Combination | 70 | Prostate | 94 | Diet and exercise guide | Wait list |  | Effectiveness (home intervention) | During treatment (ADT) | Y | Y | Y |
| Rao et al.; 2012 (64) | | SC RCT; 6 mo / 6 mo | Exercise | 65 | Breast | 10 | Resistance / aerobic training | Standard care |  | Efficacy (supervised bootcamp) | During treatment | Y |  |  |
| Villarini et al.; 2012 (65) | | SC RCT; NR | Dietary | 50 | Breast | 96 | Mediterranean | Standard care |  | Efficacy (cooking class, group dinner) | During treatment | Y | Y | Y |
| Von Gruenigen et al; 2012 (86) | | SC, CO, RCT ; 6 mo / 12 mo | Combination | 58 | Endometrial | 75 | Diet and exercise counselling | Wait list |  | Efficacy (intensive counselling) | Post treatment |  | Y | Y |
| DeNysschen et al.; 2011 (66) | | MC RCT; 8-12 mo / NR | Exercise | 50 | Breast | 100 | Aerobic exercise during entire chemo | Standard care | Aerobic exercise late chemotherapy | Effectiveness (individual trainer designed home program) | During and post treatment |  | Y |  |
| Guinan et al.; 2013 (67) | | SC, RCT; 8 wks / 3 mo | Exercise | 48 | Breast | 26 | Aerobic exercise | Standard care |  | Efficacy (supervise intervention + home program) | Post treatment |  |  | Y |
| Pakiz et al; 2011(88) | | SC, RCT; 16 wks / 16 wks | Combination | 56 | Breast | 68 | Low-cal and aerobic exercise counselling | Wait list |  | Efficacy (behavioural counselling) | Post treatment | Y | Y | Y |
| Flynn et al.; 2010 (68) | | MC, RCT; 8 wks / 44 wks *report 8 wks | Dietary | 59 | Breast | 28 | Plant-based olive oil diet | NCI diet |  | Efficacy (food diaries, excl. non-compliant pt) | Post treatment | Y |  | Y |
| Thomson et al.; 2010 (69) | | SC RCT; 6 mo / 24 wks | Dietary | 56 | Breast | 40 | Low-fat (ACS) | Low-carb diet (modified Atkins) |  | Effectiveness (counselling, printed materials) | Post treatment | Y | Y |  |
| Irwin et al; 2009 (70) | | SC RCT; 6 mo / 6 mo | Exercise | 56 | Breast | 75 | Aerobic exercise | Standard care |  | Efficacy (supervised gym + home program) | Post treatment | Y | Y | Y |
| Ligibel et al.; 2009 (71) | | SC RCT; 16 wks / 16 wks | Exercise | 53 | Breast | 101 | Resistance / aerobic training | Standard care |  | Efficacy (supervised resistance, home aerobic) | Post treatment | Y | Y | Y |
| Morey et al.; 2009 (72) | | MC, CO, RCT; 12 mo / 12 mo | Combination | 73 | Breast, colorectal, prostate | 641 | Resistance / aerobic exercise + low-fat, phyto rich diet | Standard care |  | Effectiveness (telephone based intervention with mailed materials) | Long-term post treatment | Y | Y |  |
| Rogers et al.; 2009 (73) | | MC, RCT; 12 wks / 8 mo | Exercise | 53 | Breast | 41 | Resistance / aerobic exercise vs standard care | Standard care |  | Efficacy (group + indiv. sessions, counseling) | Post treatment | Y |  |  |
| Demark-Wahnefried et al; 2008 (74) | | MC RCT; 6 mo / 6 mo | Combination | 42 | Breast | 90 | Resistance / aerobic exercise + low-fat, phyto rich diet | Standard care (Ca rich diet) | Exercise | Effectiveness (tailored print material and phone counseling) | During treatment (pre-operative) | Y | Y | Y |
| Von Gruenigen et al; 2008 (87) | | SC, RCT; 6 mo / 12 mo | Combination | 55 | Endometrial | 45 | Dietary and exercise counselling | Standard care |  | Efficacy (counselling) | Post treatment |  | Y |  |
| Matthews et al.; 2007 (75) | | MC RCT; 12 wks / 12 wks | Exercise | 54 | Breast | 36 | Aerobic exercise, walking program | Standard care |  | Effectiveness (home-based program, phone intervention) | Post treatment |  | Y |  |
| Pierce et al.; 2007 (76) | | MC RCT; 6 y / 6 y | Dietary | 53 | Breast | 3088 | Low-fat, high phyto and fiber diet | NCI diet |  | Effectiveness (phone intervention, cooking classes) | Post treatment |  | Y |  |
| Shaw et al.; 2007 (77) | | SC RCT; 24 wks / 24 wks | Dietary | 65 | Breast | 51 | Low-fat diet | Standard care | Low-calorie | Effectiveness (nutritionist advice and printed materials) | Post treatment | Y | Y |  |
| Chlebowski et al.; 2007 (20) | | MC, RCT; 60 mo / 60 mo | Dietary | 59 | Breast | 2437 | Low-fat diet | Standard care |  | Efficacy (individual biwkly counselling, dietician, group sessions) | During treatment | Y | Y |  |
| Herrero et al.; 2006 (78) | | SC RCT; 8 wks / 8 wks | Exercise | 51 | Breast | 16 | Aerobic / resistance exercise | Standard care |  | Efficacy (supervised exercise) | Post treatment |  | Y |  |
| Schmitz et al.; 2005 (79) | | SC RCT; 12mo / 12 mo | Exercise | 53 | Breast | 79 | Resistance | Standard care |  | Efficacy (supervised exercise) | Post treatment | Y | Y | Y |
| Jen et al.; 2004 (80) | | SC RCT; 12 mo / 12 mo | Dietary | Range 36-70 | Breast | 48 | Weight watchers | NCI diet | individualized diet; comprehensive (WW + diet) | Efficacy (weekly dietitian meetings, attendance to WW) | Post treatment | Y | Y |  |
| Courneya et al.; 2003 (24) | | SC, CO, RCT; 15 wks / 15 wks | Exercise | 59 | Breast | 53 | Aerobic exercise | Standard care |  | Efficacy (supervised exercise) | Post treatment | Y | Y |  |
| Burnham et al.; 2002 (81) | | SC RCT; 10 wks / 10 wks | Exercise | 54 | Breast, colorectal | 18 | Aerobic exercise | Standard care |  | Efficacy (supervised exercise) | Post treatment |  | Y |  |
| Segal et al.; 2001 (82) | | SC RCT; 26 wks / 26 wks | Exercise | 51 | Breast | 123 | Supervised aerobic | Standard care | Self-directed aerobic | Efficacy vs effective (supervised vs self-directed) | During treatment |  | Y |  |
| ***Abbreviations.*** SC= single centre, MC= multi centre, CO=cross-over, PM = post-menopausal, BMI = body mass index, WT=weight, WC = waist circumference, NR = not reported | | | | | | | | | | | | | | |

**Risk of Bias Assessments of Studies Included in NMAs**

Findings of risk of bias assessment are summarized below. Regarding coloring in the table, red cells indicate high risk of bias, yellow cells indicate unclear risk of bias, and green cells indicate low risk of bias.

| **Study** | **Random sequence generation** | **Allocation concealment** | **Blinding (participants and personnel)** | **Blinding (outcome assessment)** | **Incomplete outcome data** | **Selective reporting** | **Intention to treat** |
| --- | --- | --- | --- | --- | --- | --- | --- |
| Freedland et al.; 2019 (45) |  |  |  |  |  |  |  |
| McNeil et al.; 2019 (46) |  |  |  |  |  |  |  |
| Mijwel et al.; 2019 (47) |  |  |  |  |  |  |  |
| Bourke et al.; 2018 (48) |  |  |  |  |  |  |  |
| Dieli-Conwright et al.; 2018 (49) |  |  |  |  |  |  |  |
| Henning et al.; 2018 (50) |  |  |  |  |  |  |  |
| Toohey et al.; 2018 (51) |  |  |  |  |  |  |  |
| Skouroliakou et al.; 2018 (106) |  |  |  |  |  |  |  |
| Braakhuis et al.; 2017 (53) |  |  |  |  |  |  |  |
| Demark-Wahnefried et al.; 2017 (54) |  |  |  |  |  |  |  |
| Arikawa et al.; 2017 (105) |  |  |  |  |  |  |  |
| Wall et al.; 2017 (107) |  |  |  |  |  |  |  |
| Roveda et al.; 2017 (108) |  |  |  |  |  |  |  |
| Thomas et al.; 2017 (110) |  |  |  |  |  |  |  |
| Hojan et al.; 2017 (111) |  |  |  |  |  |  |  |
| Kim et al.; 2017 (112) |  |  |  |  |  |  |  |
| Rogers et al.; 2016 (114) |  |  |  |  |  |  |  |
| Greenlee et al; 2016 (115) |  |  |  |  |  |  |  |
| Cantarero-Vallaneuva et al.; 2015 (116) |  |  |  |  |  |  |  |
| Befort et al.; 2016 (117) |  |  |  |  |  |  |  |
| Winters-Stone et al.; 2016 (118) |  |  |  |  |  |  |  |
| Reeves et al.; 2017 (119) |  |  |  |  |  |  |  |
| Courneya et al.; 2016 (120) |  |  |  |  |  |  |  |
| Brown et al.; 2017 (121) |  |  |  |  |  |  |  |
| Dolan et al; 2016 (89) |  |  |  |  |  |  |  |
| Gilbert et al; 2016 (90) |  |  |  |  |  |  |  |
| Harrigan et al; 2016 (91) |  |  |  |  |  |  |  |
| Hvid et al; 2016 (92) |  |  |  |  |  |  |  |
| Lahart et al; 2016 (122) |  |  |  |  |  |  |  |
| Sheppard et al; 2016 (94) |  |  |  |  |  |  |  |
| Brown et al; 2015 (95) |  |  |  |  |  |  |  |
| Casla et al; 2015 (96) |  |  |  |  |  |  |  |
| Cornette et al; 2015 (97) |  |  |  |  |  |  |  |
| Edvardsen et al; 2015 (98) |  |  |  |  |  |  |  |
| Kampshoff et al; 2015 (100) |  |  |  |  |  |  |  |
| Nilsen et al; 2015 (101) |  |  |  |  |  |  |  |
| Swisher et al; 2015 (102) |  |  |  |  |  |  |  |
| Travier et al; 2015 (103) |  |  |  |  |  |  |  |
| Van Vulpen et al; 2015 (104) |  |  |  |  |  |  |  |
| Cormie et al; 2015 (83) |  |  |  |  |  |  |  |
| Backman et al; 2014 (60) |  |  |  |  |  |  |  |
| Cho et al.; 2014 (56) |  |  |  |  |  |  |  |
| Courneya et al.; 2014 (129) |  |  |  |  |  |  |  |
| Demark-Wahnefried et al.; 2014 (58) |  |  |  |  |  |  |  |
| Goodwin et al.; 2014 (59) |  |  |  |  |  |  |  |
| Greenlee et al.; 2013 (61) |  |  |  |  |  |  |  |
| Scott et al.; 2013 (62) |  |  |  |  |  |  |  |
| Wright et al; 2013 (84) |  |  |  |  |  |  |  |
| Demark-Wahnefried et al.; 2012 (32) |  |  |  |  |  |  |  |
| Oneill et al; 2012 (85) |  |  |  |  |  |  |  |
| Rao et al.; 2012 (64) |  |  |  |  |  |  |  |
| Villarini et al.; 2012 (65) |  |  |  |  |  |  |  |
| Von Gruenigen et al; 2012 (86) |  |  |  |  |  |  |  |
| DeNysschen et al.; 2011 (66) |  |  |  |  |  |  |  |
| Guinan et al.; 2013 (67) |  |  |  |  |  |  |  |
| Pakiz et al; 2011(88) |  |  |  |  |  |  |  |
| Flynn et al.; 2010 (68) |  |  |  |  |  |  |  |
| Thomson et al.; 2010 (69) |  |  |  |  |  |  |  |
| Irwin et al; 2009 (70) |  |  |  |  |  |  |  |
| Ligibel et al.; 2009 (71) |  |  |  |  |  |  |  |
| Morey et al.; 2009 (72) |  |  |  |  |  |  |  |
| Rogers et al.; 2009 (73) |  |  |  |  |  |  |  |
| Demark-Wahnefried et al; 2008 (74) |  |  |  |  |  |  |  |
| Von Gruenigen et al; 2008 (87) |  |  |  |  |  |  |  |
| Matthews et al.; 2007 (75) |  |  |  |  |  |  |  |
| Pierce et al.; 2007 (76) |  |  |  |  |  |  |  |
| Shaw et al.; 2007 (77) |  |  |  |  |  |  |  |
| Chlebowski et al.; 2007 (20) |  |  |  |  |  |  |  |
| Herrero et al.; 2006 (78) |  |  |  |  |  |  |  |
| Schmitz et al.; 2005 (79) |  |  |  |  |  |  |  |
| Jen et al.; 2004 (80) |  |  |  |  |  |  |  |
| Courneya et al.; 2003 (24) |  |  |  |  |  |  |  |
| Burnham et al.; 2002 (81) |  |  |  |  |  |  |  |
| Segal et al.; 2001 (82) |  |  |  |  |  |  |  |
