# Supplementary material for: Optimising weight-loss interventions in cancer patients—A systematic review and network meta-analysis
Source: PLoS One. 2021 Feb 4;16(2):e0245794. doi: 10.1371/journal.pone.0245794 (PMC7861370; doi:10.1371/journal.pone.0245794)
Supplement: S6 Text — (DOCX) [file pone.0245794.s006.docx]

**S6 Text: Model Fit Statistics from Network Meta-Analyses**

The adequacy of fit for a network meta-analysis model can be evaluated by comparing the number of unconstrained data points in the data set (i.e. the total number of treatment arms across studies) to the total residual deviance of the corresponding analysis; they should be of similar value. To compare the fit of competing models, their deviance information criteria (DIC) values can be compared, with smaller values being preferable and differences of 5 points or more reflecting an important difference.

| **Model** | **# unconstrained data points *** | **Total residual deviance** | **Between-study SD (95% CrI)** | **DIC** |
| --- | --- | --- | --- | --- |
| **Change in Body Weight** | | | | |
| RE consistency | 143 (84 + 59) | 149.72 | 1.254 (0.848-1.667) | 342.1 |
| RE unrelated means | 143 (84 + 59) | 149.83 | 1.222 (0.820-1.644) | 347.7 |
| FE consistency | 143 (84 + 59) | 209.48 | NA | 468.5 |
| **Change in BMI** | | | | |
| RE consistency | 101 (55 + 46) | 97.67 | 0.139 (0.005-0.450) | 126.2 |
| RE unrelated means | 101 (55 + 46) | 97.58 | 0.117 (0.003-0.388) | 128.7 |
| FE consistency | 101 (55 + 46) | 98.40 | NA | 130.1 |
| **Change in Waist Circumference** | | | | |
| RE consistency | 69 (41 + 28) | 65.31 | 0.878 (0.201-1.608) | 232.0 |
| RE unrelated means | 69 (41 + 28) | 65.45 | 0.853 (0.154-1.595) | 232.4 |
| FE consistency | 69 (41 + 28) | 72.77 | NA | 240.2 |

*: Number of unconstrained data points = number of arms with reported mean change + number of study arms with reported pre- and post-intervention data

ǂ: Posterior mean estimate for the correlation between pre- and post-intervention outcomes in studies which did not report the changes and corresponding SDs.
