# Supplementary material for: Optimising weight-loss interventions in cancer patients—A systematic review and network meta-analysis
Source: PLoS One. 2021 Feb 4;16(2):e0245794. doi: 10.1371/journal.pone.0245794 (PMC7861370; doi:10.1371/journal.pone.0245794)

**S7 Text: Additional NMA Findings**

**A) League Tables of all Pairwise Comparisons from NMA**

**Changes in Body Weight**

League tables of estimated posterior median pairwise differences in weight change (kg) with credible intervals / 2.5% and 97.5% quantiles (lower triangle), and the pairwise probabilities that a treatment is better than another (upper triangle) are presented. A complete summary of estimates for efficacy from the RE consistency model assuming vague priors is displayed. Statistically significant differences between intervention categories are shown in bold, underlined font. The lower/right-most comparison for each comparison is the reference treatment.

The first table presents results from comparisons at the group level, while the second presents findings at the intervention level.

**Change in Body Weight (Group Level Comparisons)**

| **Combination** | 0.631 | **0.993** | **1.000** |
| --- | --- | --- | --- |
| -0.26 (-2.04 to 1.19) | **Diet** | 0.965 | **0.998** |
| **-1.82 (-3.43 to -0.50)** | -1.56 (-3.12 to 0.17) | **Exercise** | 0.914 |
| **-2.52 (-3.54 to -1.62)** | **-2.25 (-3.43 to -0.91)** | -0.69 (-1.75 to 0.49) | **Standard Care** |

**Change in Body Weight (Treatment Level Comparisons)**

| **LOWFAT/PHYTO+ AER/RES** | 0.234 | 0.358 | 0.553 | 0.462 | 0.196 | 0.163 | 0.531 | 0.531 | 0.280 | 0.561 | 0.541 | 0.474 | 0.449 | 0.353 | 0.961 | **0.991** | 0.971 | **0.999** |
| --- | --- | --- | --- | --- | --- | --- | --- | --- | --- | --- | --- | --- | --- | --- | --- | --- | --- | --- |
| 0.41 (-0.64, 2.79) | **LOWCAL+ LOWFAT+AER** | 0.677 | 0.768 | 0.749 | 0.379 | 0.387 | 0.715 | 0.716 | 0.508 | 0.743 | 0.721 | 0.672 | 0.698 | 0.583 | **0.996** | **0.999** | **0.997** | **1.000** |
| 0.14 (-0.96, 1.76) | -0.21 (-2.25, 0.92) | **LOWCAL**  **+ AER** | 0.663 | 0.607 | 0.257 | 0.223 | 0.623 | 0.622 | 0.354 | 0.657 | 0.631 | 0.564 | 0.567 | 0.440 | **0.990** | **0.998** | **0.994** | **1.000** |
| -0.04 (-2.12, 1.39) | -0.46 (-3.63, 0.68) | -0.19 (-2.61, 1.03) | **MEDIT**  **+ AER** | 0.416 | 0.198 | 0.170 | 0.484 | 0.486 | 0.283 | 0.507 | 0.492 | 0.440 | 0.417 | 0.341 | 0.882 | 0.949 | 0.898 | 0.971 |
| 0.03 (-1.18, 1.42) | -0.36 (-2.57, 0.68) | -0.10 (-1.58, 1.01) | 0.08 (-1.27, 2.24) | **LOWCAL**  **+ AER/RES** | 0.208 | 0.173 | 0.557 | 0.557 | 0.293 | 0.590 | 0.567 | 0.497 | 0.479 | 0.373 | **0.976** | **0.995** | **0.984** | **1.000** |
| 0.60 (-0.52, 4.70) | 0.13 (-1.25, 3.24) | 0.39 (-0.74, 4.12) | 0.63 (-0.55, 5.58) | 0.54 (-0.56, 4.48) | **LOWCARB + AER** | 0.529 | 0.752 | 0.754 | 0.615 | 0.772 | 0.754 | 0.724 | 0.746 | 0.669 | **0.997** | **0.999** | **0.998** | **1.000** |
| 0.64 (-0.38, 3.02) | 0.11 (-1.20, 1.88) | 0.41 (-0.57, 2.49) | 0.68 (-0.43, 3.92) | 0.58 (-0.41, 2.85) | -0.02 (-2.80, 1.51) | **LOWFAT**  **+ AER** | 0.772 | 0.773 | 0.600 | 0.795 | 0.776 | 0.736 | 0.771 | 0.666 | **1.000** | **1.000** | **1.000** | **1.000** |
| -0.07 (-2.15, 1.79) | -0.60 (-3.59, 1.23) | -0.30 (-2.57, 1.46) | 0.04 (-2.08, 2.33) | -0.14 (-2.22, 1.65) | -0.79 (-5.48, 1.15) | -0.80 (-3.85, 1.02) | **LOWFAT**  **+ PHYTO** | 0.506 | 0.242 | 0.533 | 0.511 | 0.444 | 0.424 | 0.316 | 0.887 | 0.953 | 0.905 | **0.976** |
| -0.07 (-2.17, 1.85) | -0.60 (-3.54, 1.22) | -0.30 (-2.58, 1.50) | 0.04 (-2.11, 2.43) | -0.13 (-2.23, 1.70) | -0.80 (-5.35, 1.14) | -0.80 (-3.80, 1.01) | 0.00 (-1.91, 2.00) | **LOWCAL**  **+ LOWFAT** | 0.245 | 0.530 | 0.505 | 0.440 | 0.422 | 0.316 | 0.888 | 0.952 | 0.905 | **0.976** |
| 0.53 (-1.20, 2.79) | -0.02 (-1.84, 1.80) | 0.31 (-1.33, 2.29) | 0.57 (-1.28, 3.73) | 0.48 (-1.20, 2.57) | -0.29 (-3.34, 1.55) | -0.22 (-2.05, 1.41) | 0.41 (-0.70, 3.36) | 0.41 (-0.75, 3.36) | **MEDIT** | 0.787 | 0.763 | 0.706 | 0.740 | 0.596 | **0.994** | **0.997** | **0.996** | **1.000** |
| -0.14 (-2.04, 1.56) | -0.67 (-3.45, 1.11) | -0.37 (-2.47, 1.30) | -0.02 (-2.04, 2.12) | -0.20 (-2.13, 1.46) | -0.87 (-5.41, 1.05) | -0.88 (-3.72, 0.93) | -0.03 (-1.69, 1.53) | -0.02 (-1.96, 1.72) | -0.50 (-3.27, 0.58) | **PHYTO**  **/ PLANT** | 0.475 | 0.408 | 0.382 | 0.282 | 0.887 | 0.957 | 0.906 | **0.983** |
| -0.09 (-2.18, 1.72) | -0.62 (-3.65, 1.21) | -0.32 (-2.62, 1.43) | 0.02 (-2.13, 2.25) | -0.16 (-2.27, 1.60) | -0.81 (-5.55, 1.14) | -0.82 (-3.89, 1.01) | -0.01 (-1.46, 1.36) | 0.00 (-2.03, 1.81) | -0.43 (-3.42, 0.66) | 0.02 (-1.40, 1.46) | **NCI** | 0.433 | 0.411 | 0.309 | 0.883 | 0.949 | 0.899 | 0.974 |
| 0.06 (-1.99, 2.23) | -0.46 (-3.26, 1.43) | -0.16 (-2.35, 1.79) | 0.15 (-1.95, 2.88) | 0.01 (-2.05, 2.06) | -0.68 (-4.96, 1.28) | -0.66 (-3.52, 1.17) | 0.05 (-1.62, 2.42) | 0.05 (-1.75, 2.46) | -0.29 (-3.00, 0.99) | 0.09 (-1.47, 2.39) | 0.06 (-1.54, 2.45) | **LOWCARB** | 0.490 | 0.371 | 0.917 | 0.964 | 0.930 | **0.984** |
| 0.10 (-1.50, 1.81) | -0.47 (-2.60, 1.17) | -0.14 (-1.76, 1.44) | 0.19 (-1.59, 2.62) | 0.04 (-1.53, 1.65) | -0.71 (-4.34, 1.11) | -0.69 (-2.80, 0.96) | 0.07 (-1.26, 2.25) | 0.07 (-1.34, 2.26) | -0.33 (-2.30, 0.69) | 0.12 (-1.08, 2.16) | 0.08 (-1.18, 2.29) | 0.01 (-1.58, 1.84) | **LOWFAT** | 0.351 | 0.969 | **0.990** | **0.978** | **0.999** |
| 0.34 (-1.41, 2.67) | -0.20 (-2.27, 1.69) | 0.13 (-1.62, 2.17) | 0.41 (-1.47, 3.53) | 0.29 (-1.44, 2.48) | -0.46 (-3.78, 1.46) | -0.40 (-2.51, 1.35) | 0.24 (-0.99, 3.12) | 0.24 (-1.05, 3.10) | -0.09 (-1.99, 1.36) | 0.31 (-0.85, 3.06) | 0.26 (-0.93, 3.18) | 0.14 (-1.30, 2.71) | 0.16 (-1.00, 2.07) | **LOWCAL** | **0.979** | **0.993** | **0.985** | **0.999** |
| -1.32 (-2.53, 0.15) | **-1.88 (-3.58, -0.56)** | **-1.56 (-2.74, -0.27)** | -1.27 (-2.65, 1.12) | **-1.38 (-2.55, -0.01)** | **-2.09 (-5.45, -0.65)** | **-2.09 (-3.76, -0.87)** | -1.27 (-2.89, 0.98) | -1.27 (-2.91, 0.99) | **-1.88 (-3.55, -0.40)** | -1.19 (-2.75, 0.86) | -1.24 (-2.85, 1.04) | -1.40 (-3.16, 0.77) | -1.40  (-2.81, 0.08) | **-1.69 (-3.47, -0.06)** | **AER + RES** | 0.772 | 0.570 | **0.992** |
| **-1.73 (-3.11, -0.34)** | **-2.25 (-4.43, -0.85)** | **-1.95 (-3.45, -0.67)** | -1.63 (-3.22, 0.46) | **-1.79 (-3.14, -0.48)** | **-2.44 (-6.31, -0.88)** | **-2.47 (-4.65, -1.01)** | -1.64 (-3.44, 0.37) | -1.64 (-3.48, 0.38) | **-2.27 (-4.37, -0.62)** | -1.58 (-3.23, 0.27) | -1.62 (-3.38, 0.40) | -1.78  (-3.86, 0.20) | **-1.82 (-3.42, -0.30)** | **-2.07 (-4.28, -0.41)** | -0.34 (-1.71, 0.50) | **RES** | 0.263 | 0.800 |
| -1.40 (-2.60, 0.05) | **-1.95 (-3.68, -0.66)** | **-1.63 (-2.81, -0.39)** | -1.34 (-2.72, 0.99) | **-1.46 (-2.62, -0.14)** | **-2.15 (-5.57, -0.73)** | **-2.17 (-3.87, -0.92)** | -1.33 (-2.96, 0.86) | -1.33 (-2.98, 0.86) | **-1.96 (-3.65, -0.48)** | -1.26 (-2.81, 0.73) | -1.31 (-2.93, 0.90) | -1.47 (-3.26, 0.63) | **-1.48 (-2.87, -0.04)** | **-1.76 (-3.56, -0.18)** | -0.05 (-0.95, 0.74) | 0.26 (-0.57, 1.54) | **AER** | **0.989** |
| **-2.18 (-3.19, -0.89)** | **-2.71 (-4.35, -1.60)** | **-2.40 (-3.40, -1.35)** | -2.13 (-3.33, 0.11) | **-2.24 (-3.19, -1.11)** | **-2.89 (-6.31, -1.65)** | **-2.92 (-4.54, -1.84)** | **-2.11 (-3.58, -0.02)** | **-2.11 (-3.62, -0.02)** | **-2.72 (-4.29, -1.40)** | **-2.03 (-3.42, -0.17)** | -2.09 (-3.57, 0.02) | **-2.25 (-3.91, -0.24)** | **-2.25 (-3.48, -0.99)** | **-2.52 (-4.24, -1.09)** | **-0.84 (-1.58, -0.17)** | -0.46 (-1.33, 0.66) | **-0.77 (-1.44, -0.12)** | **SC** |

**Change in BMI**

League tables of posterior median pairwise differences in BMI change with credible intervals / 2.5% and 97.5% quantiles (lower triangle), and the pairwise probabilities that a treatment is better than another (upper triangle). A complete summary of estimates for efficacy from the RE consistency model assuming vague priors is displayed. Statistically significant differences between intervention categories are shown in bold, underlined font. The lower/right-most comparison for each comparison is the reference treatment.

The first table presents results from comparisons at the group level, while the second presents findings at the intervention level.

**Change in BMI (Group Level Comparisons)**

| **Combination** | 0.541 | 0.944 | **0.998** |
| --- | --- | --- | --- |
| -0.04 (-0.99 to 0.75) | **Diet** | 0.917 | **0.992** |
| -0.67 (-1.67 to 0.20) | -0.64 (-1.56 to 0.34) | **Exercise** | 0.777 |
| **-0.91 (-1.56 to -0.36)** | **-0.87 (-1.47 to -0.22)** | -0.23 (-0.95 to 0.49) | **Standard Care** |

**Change in BMI (Treatment Level Comparisons)**

| **LOWFAT/PHYTO+ AER/RES** | 0.224 | 0.432 | 0.581 | 0.023 | 0.015 | 0.332 | 0.353 | 0.045 | 0.584 | 0.225 | 0.074 | 0.029 | 0.952 | **0.997** | 0.577 | **1.000** |
| --- | --- | --- | --- | --- | --- | --- | --- | --- | --- | --- | --- | --- | --- | --- | --- | --- |
| 0.27 (-0.44, 1.02) | **LOWCAL**  **+ AER** | 0.641 | 0.804 | 0.096 | 0.081 | 0.542 | 0.537 | 0.216 | 0.746 | 0.445 | 0.415 | 0.174 | 0.942 | **0.984** | 0.814 | **0.987** |
| 0.08 (-0.98, 1.06) | -0.18 (-1.39, 0.93) | **MEDIT**  **+ AER** | 0.594 | 0.067 | 0.067 | 0.422 | 0.426 | 0.174 | 0.601 | 0.331 | 0.291 | 0.138 | 0.777 | 0.883 | 0.614 | 0.884 |
| -0.04 (-0.46, 0.39) | -0.31 (-1.08, 0.42) | -0.11 (-1.11, 0.93) | **LOWCAL**  **+ AER/RES** | 0.022 | 0.015 | 0.311 | 0.334 | 0.042 | 0.547 | 0.208 | 0.076 | 0.029 | 0.893 | **0.992** | 0.499 | **0.998** |
| 1.13 (0.01, 2.52) | 0.84 (-0.25, 2.33) | 0.99 (-0.19, 2.85) | 1.16 (0.01, 2.58) | **LOWCARB + AER** | 0.620 | 0.882 | 0.871 | 0.772 | 0.945 | 0.858 | 0.907 | 0.737 | **0.998** | **1.000** | **0.992** | **1.000** |
| 0.97 (0.07, 1.69) | 0.66 (-0.20, 1.63) | 0.83 (-0.17, 2.17) | 1.01 (0.07, 1.76) | -0.15 (-1.40, 0.81) | **LOWFAT**  **+ AER** | 0.892 | 0.873 | 0.732 | 0.956 | 0.862 | 0.934 | 0.682 | **1.000** | **1.000** | **0.998** | **1.000** |
| 0.21 (-0.82, 1.18) | -0.06 (-1.32, 1.06) | 0.12 (-1.20, 1.44) | 0.25 (-0.81, 1.24) | -0.87 (-2.71, 0.43) | -0.71 (-2.05, 0.36) | **LOWFAT**  **+ PHYTO** | 0.493 | 0.208 | 0.695 | 0.412 | 0.388 | 0.166 | 0.847 | 0.924 | 0.705 | 0.928 |
| 0.21 (-1.21, 1.31) | -0.06 (-1.56, 1.22) | 0.13 (-1.44, 1.60) | 0.24 (-1.15, 1.39) | -0.86 (-3.04, 0.49) | -0.70 (-2.43, 0.45) | 0.01 (-1.51, 1.43) | **LOWCAL**  **+ LOWFAT** | 0.231 | 0.670 | 0.406 | 0.405 | 0.188 | 0.814 | 0.891 | 0.684 | 0.892 |
| 0.66 (-0.10, 1.45) | 0.36 (-0.58, 1.39) | 0.55 (-0.56, 1.88) | 0.69 (-0.09, 1.50) | -0.44 (-1.84, 0.68) | -0.28 (-1.20, 0.60) | 0.40 (-0.54, 1.69) | 0.41 (-0.71, 2.02) | **MEDIT** | 0.918 | 0.724 | 0.788 | 0.431 | **0.995** | **0.999** | 0.974 | **0.999** |
| -0.09 (-0.95, 0.79) | -0.36 (-1.49, 0.69) | -0.15 (-1.36, 1.03) | -0.05 (-0.93, 0.86) | -1.19 (-2.95, 0.21) | -1.04 (-2.18, 0.13) | -0.27 (-1.48, 0.84) | -0.24 (-1.57, 0.99) | -0.73 (-1.86, 0.28) | **PHYTO**  **/ PLANT** | 0.218 | 0.160 | 0.050 | 0.685 | 0.843 | 0.469 | 0.846 |
| 0.34 (-0.60, 1.28) | 0.07 (-1.02, 1.16) | 0.25 (-0.98, 1.65) | 0.38 (-0.57, 1.34) | -0.75 (-2.39, 0.50) | -0.58 (-1.77, 0.46) | 0.12 (-1.01, 1.39) | 0.13 (-1.12, 1.63) | -0.29 (-1.38, 0.64) | 0.40 (-0.59, 1.62) | **LOWCARB** | 0.497 | 0.220 | 0.922 | 0.969 | 0.810 | 0.972 |
| 0.37 (-0.17, 0.81) | 0.08 (-0.72, 0.86) | 0.27 (-0.72, 1.40) | 0.40 (-0.18, 0.90) | -0.76 (-2.12, 0.30) | -0.60 (-1.34, 0.20) | 0.13 (-0.78, 1.18) | 0.12 (-0.94, 1.56) | -0.29 (-1.08, 0.38) | 0.43 (-0.37, 1.34) | 0.00 (-0.82, 0.88) | **LOWFAT** | 0.118 | **0.996** | **0.999** | 0.963 | **1.000** |
| 0.73 (-0.03, 1.51) | 0.44 (-0.47, 1.46) | 0.65 (-0.46, 1.97) | 0.78 (-0.03, 1.61) | -0.35 (-1.74, 0.69) | -0.21 (-1.08, 0.65) | 0.50 (-0.48, 1.71) | 0.49 (-0.63, 2.04) | 0.07 (-0.82, 0.98) | 0.79 (-0.12, 1.96) | 0.35 (-0.56, 1.45) | 0.37 (-0.24, 1.06) | **LOWCAL** | **0.998** | **0.999** | **0.989** | **1.000** |
| -0.29 (-0.68, 0.05) | -0.57 (-1.31, 0.15) | -0.39 (-1.37, 0.68) | -0.25 (-0.70, 0.17) | **-1.42 (-2.76, -0.39)** | **-1.26 (-1.95, -0.50)** | -0.51 (-1.44, 0.53) | -0.52 (-1.59, 0.87) | **-0.95 (-1.70, -0.25)** | -0.21 (-1.04, 0.67) | -0.65 (-1.56, 0.27) | **-0.65 (-1.07, -0.21)** | **-1.03 (-1.76, -0.35)** | **AER + RES** | 0.929 | 0.063 | **0.975** |
| **-0.50 (-0.92, -0.18)** | **-0.79 (-1.54, -0.07)** | -0.60 (-1.54, 0.45) | **-0.46 (-0.94, -0.09)** | **-1.64 (-3.00, -0.54)** | **-1.48 (-2.17, -0.64)** | -0.72 (-1.65, 0.30) | -0.73 (-1.82, 0.67) | **-1.17 (-1.94, -0.46)** | -0.43 (-1.28, 0.43) | -0.86 (-1.77, 0.05) | **-0.87 (-1.30, -0.42)** | **-1.24 (-2.00, -0.54)** | -0.21 (-0.55, 0.08) | **RES** | **0.013** | 0.491 |
| -0.04 (-0.53, 0.32) | -0.33 (-1.08, 0.41) | -0.14 (-1.08, 0.95) | 0.00 (-0.54, 0.43) | **-1.17 (-2.51, -0.18)** | **-1.00 (-1.70, -0.29)** | -0.27 (-1.19, 0.80) | -0.28 (-1.34, 1.17) | -0.71 (-1.45, 0.01) | 0.04 (-0.82, 0.93) | -0.41 (-1.30, 0.54) | -0.40 (-0.86, 0.04) | **-0.77 (-1.52, -0.11)** | 0.25 (-0.08, 0.55) | **0.47 (0.07, 0.80)** | **AER** | **1.000** |
| **-0.50 (-0.89, -0.24)** | **-0.80 (-1.51, -0.09)** | -0.61 (-1.52, 0.44) | **-0.47 (-0.90, -0.13)** | **-1.64 (-2.98, -0.66)** | **-1.47 (-2.13, -0.77)** | -0.74 (-1.63, 0.29) | -0.74 (-1.79, 0.67) | **-1.17 (-1.90, -0.52)** | -0.43 (-1.26, 0.42) | -0.87 (-1.74, 0.03) | **-0.87 (-1.25, -0.50)** | **-1.24 (-1.95, -0.63)** | **-0.22 (-0.46, 0.00)** | 0.00 (-0.27, 0.22) | **-0.47 (-0.72, -0.21)** | **SC** |

**Change in Waist Circumference**

League tables of posterior median pairwise differences in waist circumference change (cm) with credible intervals / 2.5% and 97.5% quantiles (lower triangle), and the pairwise probabilities that a treatment is better than another (upper triangle). A complete summary of estimates for efficacy from the RE consistency model assuming vague priors is displayed. Statistically significant differences between intervention categories are shown in bold, underlined font. The lower/right-most comparison for each comparison is the reference treatment.

The first table presents results from comparisons at the group level, while the second presents findings at the intervention level.

**Change in Waist Circumference (Group Level Comparisons)**

| **Combination** | 0.574 | 0.825 | **1.000** |
| --- | --- | --- | --- |
| -0.19  (-2.30 to 1.85) | **Diet** | 0.710 | **0.997** |
| -0.72  (-2.45 to 0.82) | -0.53  (-2.53 to 1.44) | **Exercise** | **0.996** |
| **-2.51**  **(-3.81 to -1.34)** | **-2.32**  **(-4.02 to -0.69)** | **-1.78**  **(-2.89 to -0.64)** | **Standard Care** |

**Change in Waist Circumference (Treatment Level Comparisons)**

| **LOWFAT/PHYTO + AER/RES** | 0.337 | 0.518 | 0.566 | 0.657 | 0.468 | 0.602 | 0.516 | 0.574 | 0.605 | 0.576 | 0.535 | 0.765 | 0.833 | 0.799 | **0.996** |
| --- | --- | --- | --- | --- | --- | --- | --- | --- | --- | --- | --- | --- | --- | --- | --- |
| 0.16 (-1.02, 2.67) | **LOWCAL +**  **LOWFAT + AER** | 0.674 | 0.715 | 0.789 | 0.649 | 0.706 | 0.647 | 0.687 | 0.705 | 0.679 | 0.651 | 0.885 | 0.912 | 0.905 | **1.000** |
| -0.01 (-2.06, 1.84) | -0.18 (-2.92, 0.99) | **LOWCAL + AER** | 0.549 | 0.636 | 0.451 | 0.588 | 0.502 | 0.559 | 0.590 | 0.561 | 0.521 | 0.743 | 0.817 | 0.782 | **0.992** |
| -0.05 (-2.40, 1.51) | -0.26 (-3.22, 0.77) | -0.03 (-2.29, 1.65) | **MEDIT + AER** | 0.590 | 0.398 | 0.554 | 0.466 | 0.521 | 0.556 | 0.528 | 0.486 | 0.706 | 0.787 | 0.745 | **0.990** |
| -0.15 (-2.53, 1.06) | -0.44 (-3.15, 0.47) | -0.13 (-2.45, 1.25) | -0.07 (-2.06, 1.40) | **LOWCAL**  **+ AER/RES** | 0.300 | 0.499 | 0.390 | 0.458 | 0.501 | 0.472 | 0.422 | 0.629 | 0.748 | 0.677 | **1.000** |
| 0.02 (-1.60, 1.89) | -0.14 (-2.20, 0.94) | 0.03 (-1.57, 2.14) | 0.08 (-1.26, 2.41) | 0.21 (-0.79, 2.39) | **LOWFAT**  **+ AER** | 0.628 | 0.538 | 0.601 | 0.630 | 0.599 | 0.557 | 0.804 | 0.868 | 0.843 | **1.000** |
| -0.30 (-3.12, 2.08) | -0.63 (-3.73, 1.57) | -0.26 (-3.07, 2.20) | -0.16 (-2.74, 2.33) | 0.00 (-2.23, 2.27) | -0.37 (-3.07, 1.85) | **LOWFAT**  **+ PHYTO** | 0.370 | 0.449 | 0.509 | 0.465 | 0.398 | 0.617 | 0.701 | 0.654 | **0.983** |
| -0.04 (-2.32, 2.34) | -0.39 (-2.68, 1.65) | -0.01 (-2.36, 2.52) | 0.10 (-2.15, 2.71) | 0.29 (-1.76, 2.59) | -0.10 (-2.26, 2.02) | 0.11 (-1.09, 2.51) | **MEDIT** | 0.592 | 0.640 | 0.596 | 0.531 | 0.735 | 0.802 | 0.773 | **0.999** |
| -0.22 (-2.77, 2.13) | -0.55 (-3.28, 1.60) | -0.18 (-2.75, 2.27) | -0.06 (-2.49, 2.41) | 0.11 (-2.01, 2.35) | -0.28 (-2.67, 1.88) | 0.03 (-1.47, 1.94) | -0.07 (-2.01, 1.14) | **PHYTO / PLANT** | 0.572 | 0.513 | 0.440 | 0.659 | 0.741 | 0.698 | **0.994** |
| -0.31 (-3.17, 2.06) | -0.64 (-3.79, 1.57) | -0.28 (-3.16, 2.18) | -0.18 (-2.86, 2.29) | 0.00 (-2.28, 2.27) | -0.38 (-3.11, 1.86) | 0.00 (-1.82, 1.74) | -0.13 (-2.48, 1.04) | -0.05 (-1.62, 1.10) | **NCI** | 0.456 | 0.387 | 0.608 | 0.694 | 0.647 | **0.980** |
| -0.23 (-3.02, 2.35) | -0.55 (-3.61, 1.69) | -0.19 (-3.01, 2.46) | -0.08 (-2.76, 2.64) | 0.08 (-2.23, 2.62) | -0.29 (-2.97, 2.05) | 0.02 (-1.68, 2.16) | -0.07 (-2.27, 1.32) | -0.01 (-1.76, 1.71) | 0.03 (-1.55, 2.09) | **LOWCARB** | 0.431 | 0.643 | 0.719 | 0.680 | **0.984** |
| -0.10 (-2.59, 2.46) | -0.43 (-3.00, 1.74) | -0.06 (-2.56, 2.63) | 0.04 (-2.34, 2.76) | 0.22 (-1.95, 2.70) | -0.16 (-2.51, 2.11) | 0.08 (-1.24, 2.40) | -0.02 (-1.64, 1.42) | 0.04 (-1.29, 1.97) | 0.09 (-1.14, 2.40) | 0.05 (-1.31, 2.06) | **LOWFAT** | 0.701 | 0.771 | 0.736 | **0.996** |
| -0.64 (-2.55, 1.29) | -0.97 (-2.96, 0.55) | -0.59 (-2.57, 1.51) | -0.49 (-2.35, 1.73) | -0.30 (-1.99, 1.48) | -0.69 (-2.44, 0.90) | -0.31 (-2.41, 2.02) | -0.58 (-2.54, 1.25) | -0.39 (-2.43, 1.64) | -0.28 (-2.42, 2.12) | -0.39 (-2.60, 1.95) | -0.52 (-2.63, 1.51) | **AER + RES** | 0.657 | 0.576 | **0.999** |
| -0.85 (-3.12, 0.94) | -1.19 (-3.62, 0.46) | -0.82 (-3.10, 1.12) | -0.72 (-2.78, 1.27) | -0.53 (-2.24, 1.00) | -0.93 (-2.98, 0.70) | -0.55 (-2.75, 1.66) | -0.84 (-3.00, 1.09) | -0.65 (-2.79, 1.36) | -0.54 (-2.77, 1.72) | -0.63 (-3.04, 1.63) | -0.77 (-3.13, 1.28) | -0.14 (-1.72, 0.88) | **RES** | 0.389 | **0.993** |
| -0.72 (-2.69, 1.07) | -1.07 (-3.11, 0.47) | -0.68 (-2.67, 1.26) | -0.58 (-2.43, 1.44) | -0.37 (-1.98, 1.14) | -0.79 (-2.53, 0.72) | -0.40 (-2.45, 1.81) | -0.68 (-2.62, 1.13) | -0.50 (-2.48, 1.47) | -0.38 (-2.47, 1.86) | -0.48 (-2.68, 1.78) | -0.63 (-2.73, 1.36) | -0.05 (-1.25, 0.96) | 0.08 (-0.93, 1.46) | **AER** | **1.000** |
| **-2.54 (-4.31, -0.93)** | **-2.86 (-4.72, -1.58)** | **-2.50 (-4.29, -0.71)** | **-2.40 (-4.02, -0.53)** | **-2.20 (-3.48, -0.87)** | **-2.60 (-4.12, -1.33)** | **-2.21 (-4.07, -0.20)** | **-2.49 (-4.22, -0.92)** | **-2.31 (-4.10, -0.56)** | **-2.20 (-4.09, -0.11)** | **-2.30 (-4.34, -0.22)** | **-2.44 (-4.36, -0.66)** | **-1.91 (-2.91, -0.88)** | **-1.64 (-2.77, -0.43)** | **-1.80 (-2.72, -0.91)** | **SC** |

**B) Comparison-Adjusted Funnel Plots**

Comparison-adjusted funnel plots were prepared for each endpoint to monitor for signs of publication bias.

**Changes in Body Weight**

**
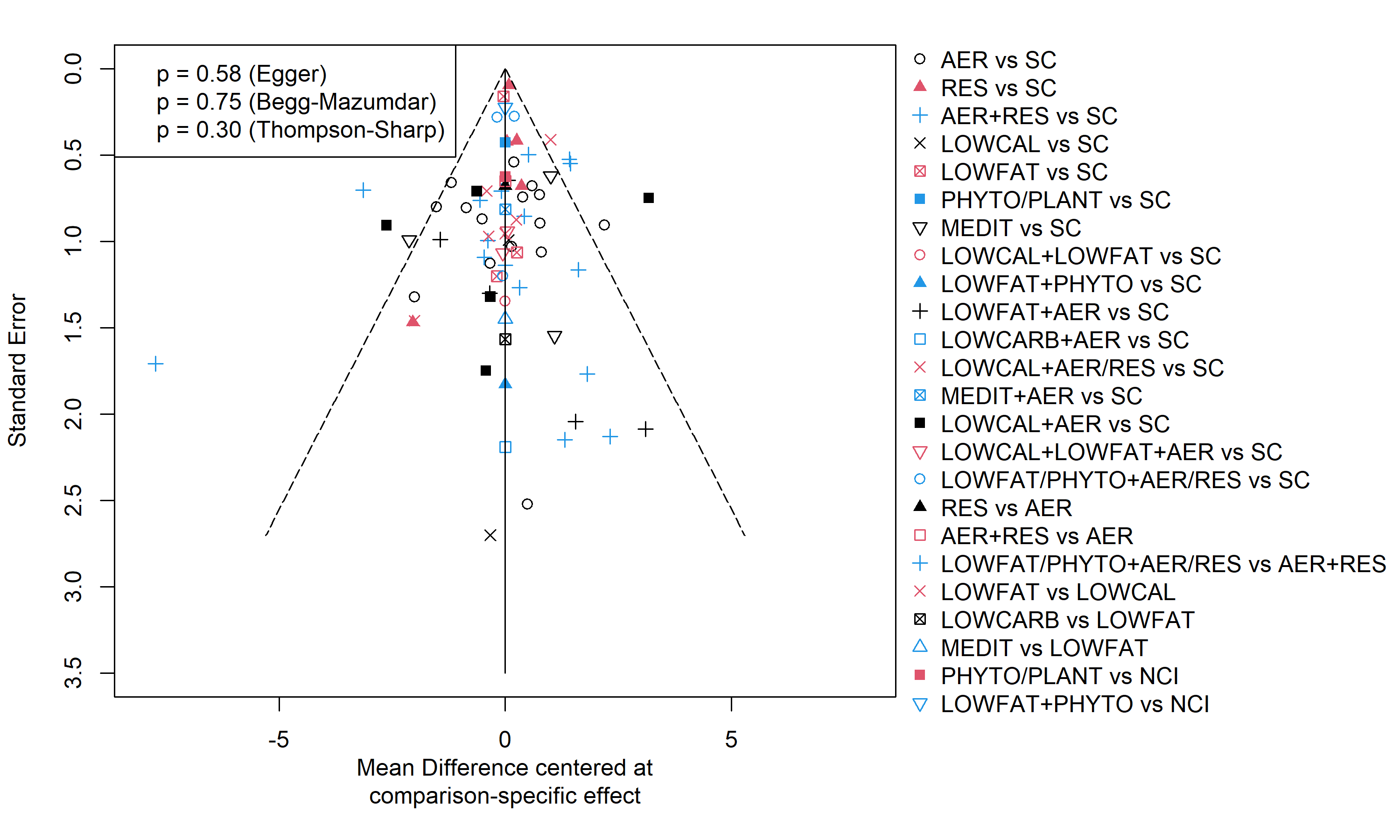
**

**Change in BMI

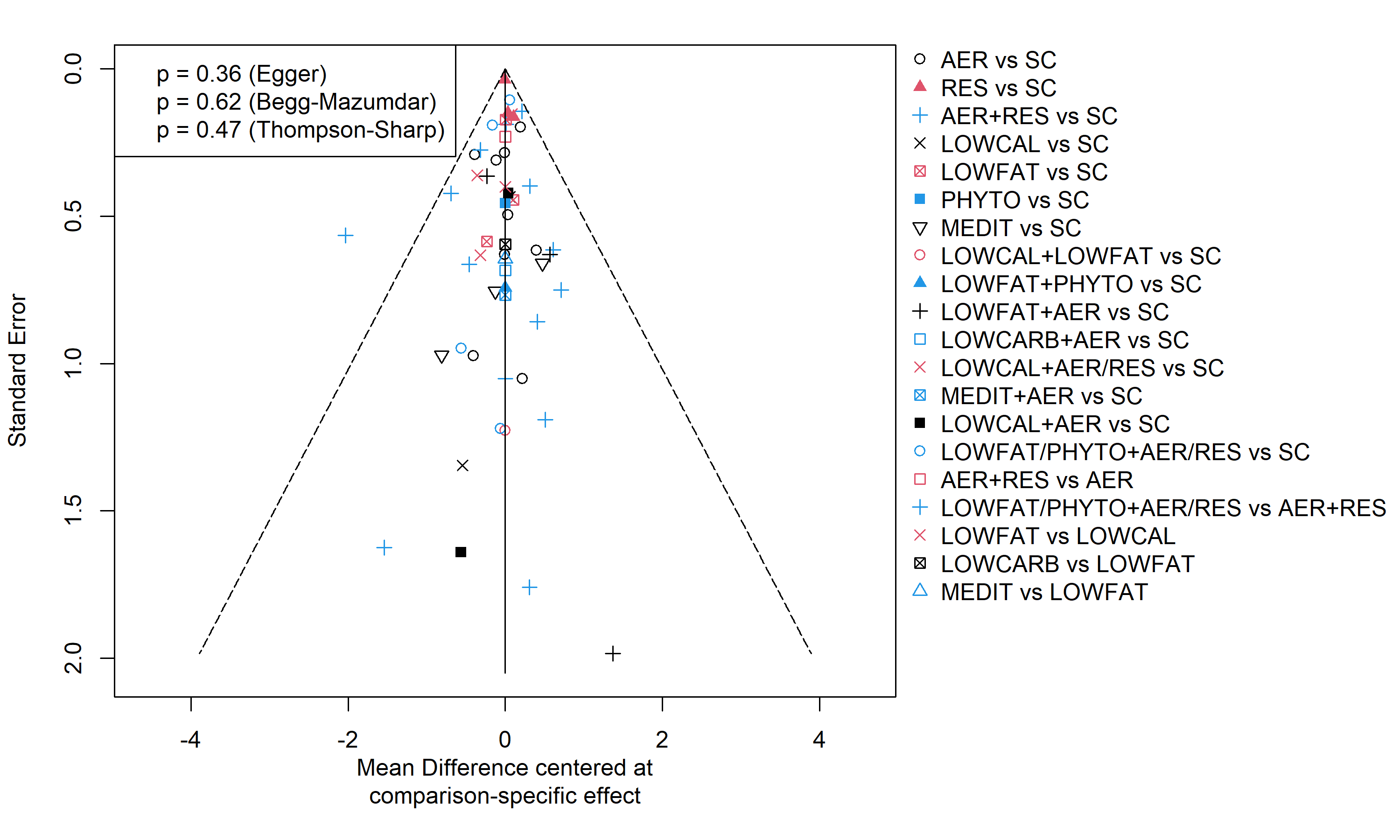
**

**Change in Waist Circumference**


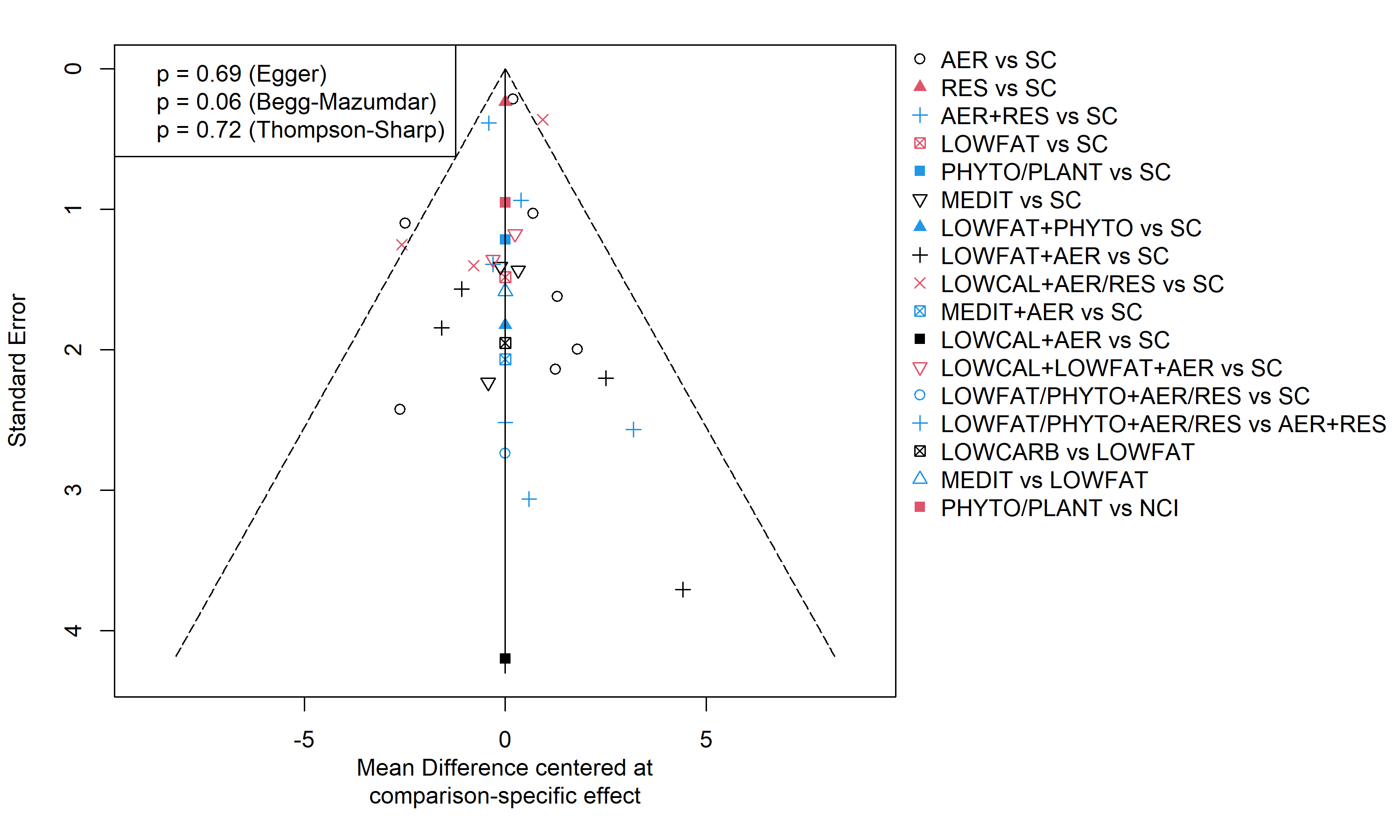

Supplement: S7 Text — (DOCX) [file pone.0245794.s007.docx]
