# Supplementary material for: Optimising weight-loss interventions in cancer patients—A systematic review and network meta-analysis
Source: PLoS One. 2021 Feb 4;16(2):e0245794. doi: 10.1371/journal.pone.0245794 (PMC7861370; doi:10.1371/journal.pone.0245794)
Supplement: S8 Text — (DOCX) [file pone.0245794.s008.docx]

**S8 Text: OpenBUGS Code Used for Network Meta-Analyses with Annotations**

**Random Effects Consistency Model**

# Normal likelihood, identity link, random effects consistency model

# Adapted from Owen et al (2015). Modified to allow trial-level data in two formats:

# mean changes (SDs) for ns studies, and pre- and post-intervention means (SDs) for ns2 studies

norm_ident_consist <- function(){ # *** PROGRAM STARTS

for (i in 1:ns){

for (k in 1:na[i]){

sesq[i,k] <- pow(se[i,k], 2) # calculate SE^2 for the mean change

}

}

for (i in (ns+1):(ns+ns2)){

for (k in 1:na[i]){

sesq[i,k] <- (pow(sd.bsl[i-ns,k], 2) + pow(sd.end[i-ns,k], 2)

- 2 * rho * sd.bsl[i-ns,k] * sd.end[i-ns,k])/N[i-ns,k]

} # calculate SE^2 for final - baseline

}

for(i in 1:(ns+ns2)){ # LOOP THROUGH STUDIES

w[i,1] <- 0 # adjustment for multi-arm trials is 0 for control

delta[i,1] <- 0 # treatment effect is 0 for control arm

mu[i] ~ dnorm(0, 0.0001) # vague priors for all trial baselines

for (k in 1:na[i]){ # LOOP THROUGH ARMS

prec[i,k] <- 1/sesq[i,k] # set precisions

y[i,k] ~ dnorm(theta[i,k], prec[i,k]) # normal likelihood

theta[i,k] <- mu[i] + delta[i,k] # model for linear predictor

dev[i,k] <- (y[i,k]-theta[i,k])*(y[i,k]-theta[i,k])*prec[i,k]

} # Deviance contribution

resdev[i] <- sum(dev[i,1:na[i]]) # summed residual deviance for this trial

for (k in 2:na[i]){ # LOOP THROUGH ARMS

delta[i,k] ~ dnorm(md[i,k], taud[i,k]) # trial-specific mean difference distributions

md[i,k] <- d[t[i,k]] - d[t[i,1]] + sw[i,k]

# mean of treat effects distributions (with multi-arm trial correction)

taud[i,k] <- tau*2*(k-1)/k

# precision of treatment effects distributions (with multi-arm trial correction)

w[i,k] <- delta[i,k] - d[t[i,k]] + d[t[i,1]] # adjustment for multi-arm RCTs

sw[i,k] <- sum(w[i,1:k-1])/(k-1) # cumulative adjustment for multi-arm trials

}

}

totresdev <- sum(resdev[]) # Total Residual Deviance

rho ~ dunif(0.5, 1)

d[1] <- 0 # treatment effect is 0 for reference treatment

D.d[1] <- 0

# Adding in hierarchical model where class of intervention is above intervention in the hierarchy

for (k in 2:4){

d[k] ~ dnorm(D.d[2], prec.d) # Treatment class: exercise interventions

}

for (k in 5:12){

d[k] ~ dnorm(D.d[3], prec.d) # Treatment class: dietary interventions

}

for (k in 13:18){

d[k] ~ dnorm(D.d[4], prec.d) # Treatment class: combination interventions

}

for (i in 2:4){

D.d[i] ~ dnorm(0, 0.01) # vague priors on treatment class effects

}

prec.d <- pow(sd.d, -2)

sd.d ~ dunif(0, 5)

sd ~ dunif(0, 5) # vague prior for between-trial SD.

tau <- pow(sd, -2) # between-trial precision = (1/between-trial SD)^2

# Output

# pairwise treatment effect for all possible pairwise comparisons, if nt>2

for (c in 1:(nt-1)) {

for (k in (c+1):nt) {

Diff[c,k] <- d[k] - d[c]

better[c,k]<- step(-Diff[c,k]) # assumes a positive change is "bad"

}

}

# ranking on relative scale

for (k in 1:nt) {

rk[k] <- rank(d[],k) # assumes a positive change is "bad"

best[k] <- equals(rk[k],1) # calculate prob that treatment k is best

for (i in 1:nt){

prk[i,k] <- equals(rk[k],i) # calculate prob of treatment k being rank i

}

}

# Outputs for the treatment groups level

for (c in 1:(4-1)) {

for (k in (c+1):4) {

Diff.grp[c,k] <- D.d[k] - D.d[c]

better.grp[c,k] <- step(-Diff.grp[c,k]) # assumes a positive change is "bad"

}

}

for (k in 1:4) {

rk.grp[k] <- rank(D.d[],k) # assumes a positive change is "bad"

best.grp[k] <- equals(rk.grp[k],1) # calculate prob that treatment class k is best

for (i in 1:4){

prk.grp[i,k] <- equals(rk.grp[k],i) # calculate prob of treatment class k being rank i

}

}

} # *** PROGRAM ENDS

**Fixed Effects Consistency Model**

# Normal likelihood, identity link, fixed effects consistency model

# Adapted from Owen et al (2015). Modified to allow trial-level data in two formats:

# mean changes (SDs) for ns studies, and pre- and post-intervention means (SDs) for ns2 studies

norm_ident_consist <- function(){ # *** PROGRAM STARTS

for (i in 1:ns){

for (k in 1:na[i]){

sesq[i,k] <- pow(se[i,k], 2) # calculate SE^2 for the mean change

}

}

for (i in (ns+1):(ns+ns2)){

for (k in 1:na[i]){

sesq[i,k] <- (pow(sd.bsl[i-ns,k], 2) + pow(sd.end[i-ns,k], 2)

- 2 * rho * sd.bsl[i-ns,k] * sd.end[i-ns,k])/N[i-ns,k]

} # calculate SE^2 for final - baseline

}

for (i in 1:(ns+ns2)){ # LOOP THROUGH STUDIES

mu[i] ~ dnorm(0, 0.01) # vague priors for all trial baselines

for (k in 1:na[i]){ # LOOP THROUGH ARMS

prec[i,k] <- 1/sesq[i,k] # set precisions

y[i,k] ~ dnorm(theta[i,k], prec[i,k]) # Normal likelihood

theta[i,k] <- mu[i] + d[t[i,k]] - d[t[i,1]] # model for linear predictor

dev[i,k] <- (y[i,k]-theta[i,k])*(y[i,k]-theta[i,k])*prec[i,k]

} # Deviance contribution

resdev[i] <- sum(dev[i,1:na[i]]) # summed residual deviance for this trial

}

totresdev <- sum(resdev[]) # Total Residual Deviance

rho ~ dunif(0.5, 1)

d[1] <- 0 # treatment effect is 0 for reference treatment

D.d[1] <- 0

# Adding in hierarchical model where class of intervention is above intervention in the hierarchy

for (k in 2:4){

d[k] ~ dnorm(D.d[2], prec.d) # Treatment class: exercise interventions

}

for (k in 5:12){

d[k] ~ dnorm(D.d[3], prec.d) # Treatment class: dietary interventions

}

for (k in 13:18){

d[k] ~ dnorm(D.d[4], prec.d) # Treatment class: combination interventions

}

for (i in 2:4){

D.d[i] ~ dnorm(0, 0.01) # vague priors on treatment class effects

}

prec.d <- pow(sd.d, -2)

sd.d ~ dunif(0,5)

# Output

# pairwise treatment effect for all possible pairwise comparisons, if nt>2

for (c in 1:(nt-1)) {

for (k in (c+1):nt) {

Diff[c,k] <- d[k] - d[c]

better[c,k]<- step(-Diff[c,k]) # assumes a positive change is "bad"

}

}

# ranking on relative scale

for (k in 1:nt) {

rk[k] <- rank(d[],k) # assumes a positive change is "bad"

best[k] <- equals(rk[k],1) # calculate prob that treatment k is best

for (i in 1:nt){

prk[i,k] <- equals(rk[k],i) # calculate prob of treatment k being rank i

}

}

# Outputs for the treatment groups level

for (c in 1:(4-1)) {

for (k in (c+1):4) {

Diff.grp[c,k] <- D.d[k] - D.d[c]

better.grp[c,k] <- step(-Diff.grp[c,k]) # assumes a positive change is "bad"

}

}

for (k in 1:4) {

rk.grp[k] <- rank(D.d[],k) # assumes a positive change is "bad"

best.grp[k] <- equals(rk.grp[k],1) # calculate prob that treatment class k is best

for (i in 1:4){

prk.grp[i,k] <- equals(rk.grp[k],i) # calculate prob of treatment class k being rank i

}

}

} # *** PROGRAM END
